# Association between individual urinary iodine concentrations in pregnant women and maternal/newborn outcomes

**DOI:** 10.1530/EC-24-0621

**Published:** 2025-01-29

**Authors:** Fernanda Bolfi, Maryan Borcsik Marum, Samantha Ellen da Silva Fonseca, Glaucia M F S Mazeto, Celia Regina Nogueira, Vania dos Santos Nunes-Nogueira

**Affiliations:** Department of Internal Medicine, São Paulo State University (UNESP), Medical School, Botucatu, São Paulo, Brazil

**Keywords:** iodine, urine, pregnancy, systematic review, meta-analysis

## Abstract

**Objective:**

To assess whether individual diagnosis of low urinary iodine concentration (UIC) in pregnant women is associated with adverse maternal and neonatal outcomes.

**Methods:**

Studies that compared pregnant women with UIC <150 μg/L and those with UIC 150–249 μg/L were systematically reviewed. MEDLINE, Embase, LILACS and CENTRAL were our source databases. Selection of studies, risk-of-bias assessment and data extraction were performed in pairs and independently. Relative risk (RR) with 95% confidence interval (CI) was calculated as an estimate of the effect of iodine <150 μg/L. Stata software was used to perform meta-analyses. The quality of evidence was determined according to the Grading of Recommendations Assessment, Development and Evaluation.

**Results:**

In total, 7000 studies were identified, of which 63 were included. With low or very low certainty of the evidence, no difference in the incidence of miscarriage (RR: 0.87, 95% CI: 0.64–1.18, 6 studies, 4855 participants), maternal hypothyroidism (RR: 1.05, 95% CI: 0.68–1.60, 10 studies, 11,773 participants), preterm birth (RR: 1.20, 95% CI: 0.97–1.48, 13 studies, 15,644 participants), stillbirths (RR: 0.79, 95% CI: 0.34–1.82, 6 studies, 3406 participants), low birth weight (RR: 1.25, 95% CI: 0.88–1.78, 10 studies, 10,775 participants) and small for gestational age (RR: 1.11, 95% CI: 0.90–1.37, 5 studies, 4266 participants) was observed between the two groups.

**Conclusion:**

In pregnant women, individual diagnosis of UIC <150 μg/L was not associated with adverse maternal and neonatal outcomes, emphasizing UIC as a limited method to assess individual iodine status during pregnancy.

## Introduction

Iodine is an essential micronutrient for life. This mineral is not produced by the human body but obtained through food and used by the thyroid gland to produce thyroid hormones, thyroxine (T4) and triiodothyronine (T3), which are fundamental in fetal development, in the metabolic regulation of cells and in the physical and neurological growth (1, 2). Unlike most other essential dietary nutrients, iodine status is not associated with socioeconomic development but rather with geography (3).

Some foods, such as fish, seafood, dairy products and vegetables grown in iodine-sufficient soil, naturally contain iodine. However, in many countries, the diet does not meet the necessary requirements for this nutrient, thus requiring the fortification of salt with iodine. Over 1.8 billion people are at risk of iodine deficiency worldwide because they do not get adequate iodine through their diet (4). The deficiency of this element is one of the most serious health problems worldwide, affecting all populations. Inadequate iodine intake leads to inadequate thyroid hormone production and consequently hypothyroidism (2). Pregnant women and children are more vulnerable groups.

Maternal iodine is the only source of iodine for fetal thyroid hormone synthesis. Pregnant women are susceptible to iodine deficiency because of increased renal clearance and additional fetal requirements. Therefore, consuming an adequate amount of iodine during pregnancy is important for fetal development (5).

Severe iodine deficiency during pregnancy leads to fetal hypothyroidism, mental impairment, goiter and increased neonatal mortality (6). When the iodine supply is sufficient, >90% of the ingested iodine is excreted in the urine, making urinary iodine concentration (UIC) an indicator of recent iodine intake or short-term iodine status. Chronic iodine deficiency is characterized by a 20% reduction in UIC excretion (7).

Considering the adverse effects of iodine deficiency, in 2007, the World Health Organization (WHO), the United Nations Children’s Fund and the Global Iodine Network (formerly known as the International Council for Control of Iodine Deficiency Disorders) proposed an evaluation criterion for the nutritional index of iodine in pregnant women and recommended a daily intake of 250 μg of iodine for pregnant and lactating women (7).

Iodine deficiency occurs when iodine intake falls below the recommended levels. For pregnant women, median UICs less than 150 μg/L define a population that has iodine deficiency. Values between 150 and 249 μg/L indicate a population with adequate iodine intake. UIC values between 250 and 499 μg/L indicate a population consumption above the required levels, and values ≥500 μg/L indicate excessive iodine intake (7).

Although a low median UIC indicates that a population is at risk of developing thyroid disorders, UIC has limited utility in assessing individual intake or status because of large variations within and between days (7). However, these variations level out in large population samples, making UIC a useful population-level indicator; however, UIC should not be used for the purposes of individual diagnosis and treatment (7). Despite these limitations, many studies have evaluated the association between individual UICs <150 μg/L during pregnancy and adverse maternal/newborn outcomes (8, 9).

Therefore, this systematic review aimed to assess whether individual diagnosis of UIC <150 μg/L in pregnant women is associated with adverse maternal and neonatal outcomes.

## Materials and methods

This systematic review was conducted according to the Joanna Briggs Institute (JBI) methodology for systematic reviews of etiology and risk and was reported according to the Preferred Reporting Items for Systematic Reviews and Meta-Analyses (10, 11). The study protocol has been registered in the International Prospective Register of Systematic Reviews under CRD42019110085.

### Eligibility criteria

We included cohort and analytical cross-sectional studies that met the ‘PECO’ structure described as follows: population (P) – we considered pregnant women without ethnicity restrictions and without the history of thyroid diseases or other chronic diseases; exposure (E) – we included studies in pregnant women with individual diagnosis of UIC <150 μg/L; comparison (C) – women with individual diagnosis of UIC of 150–249 μg/L were included in the comparison group; and outcomes (O) – adverse outcomes more relevant from the perspectives of pregnant women and newborns were selected. In addition, the outcomes are in accordance with some of the outcomes reported in a Cochrane systematic review that evaluated the iodine supplementation for women during the preconception, pregnancy and postpartum periods (2).

The maternal outcomes analyzed were spontaneous miscarriage, hypothyroidism, subclinical hypothyroidism during pregnancy, goiter, thyroid volume and mean thyroid-stimulating hormone (TSH) values.

Neonatal/infant outcomes analyzed were preterm birth, stillbirth, low birth weight (<2500 g), small for gestational age (birth weight of less than the tenth percentile for gestational age), neonatal hypothyroidism or elevated TSH, birth length, congenital malformations (including cretinism), neonatal goiter, neonatal thyroid volume, infant death (death in the first year of life), neuro- and motor development and intelligence quotient scores (2).

### Exclusion criteria

We excluded uncontrolled studies, studies in which the control group comprised non-pregnant women and studies where participants were not stratified according to UIC levels (<150 μg/L vs ≥150 or vs 150–249 μg/L).

We intended to exclude studies whose populations were predominantly from regions with severe iodine deficiency, as these populations have already been classified as at risk of developing thyroid disorders based on their median UIC (7). However, as detailed later, no studies were excluded for this reason.

### Identification of studies

#### Electronic databases

General search strategies were created for the following electronic health databases: Embase (by Elsevier), MEDLINE (by PubMed), LILACS (by Virtual Health Library) and Registry of Controlled Clinical Studies of the Cochrane Collaboration (CENTRAL – Cochrane). Eligible studies were also surveyed in SCOPUS, Web of Science and Cumulative Index to Nursing and Allied Health Literature databases.

There were no restrictions on the language or year of publication. Databases were searched on November 07, 2019, and updated on October 30, 2023. The Medical Subject Headings that were used included ‘pregnant women’, ‘urinary iodine’, ‘iodine deficiency’ and ‘hypothyroidism’. The search strategies for the primary databases are included in the supplementary data.

We used the EndNote X9 citation management software to download references and remove duplicate entries. Initial screening of abstracts and titles was performed using the free web application Rayyan QCRI (12).

### Study selection and data extraction

Two reviewers (FB and MBM) independently selected the titles and abstracts identified during the literature search. Potentially eligible studies for inclusion in the review were read completely and subsequently assessed in terms of appropriateness using the proposed PECO structure. Any disagreement during the selection process was resolved through discussion with the project coordinator (VSN-N).

For studies selected for inclusion, both reviewers used a standardized extraction form to compute all information regarding each study.

### Assessment of the risk of bias of the included studies

The risk of bias in each selected study was assessed using the revised JBI risk-of-bias tool for etiology and risk studies. Specific instruments were used for cross-sectional and cohort studies (10). Each item was evaluated in pairs and independently by three reviewers (FB, MBM and SESF).

### Synthesis and data analysis

The unit of analysis was the data published in the included studies. The outcome data were plotted in the meta-analysis using Stata Statistical Software 17 (Stata Statistical Software, Release 17; StataCorp LLC, USA). The random-effects model was chosen as the analytic model in the meta-analysis. This decision was based on our preliminary assumption that there would be heterogeneity among the populations and studies included in the analyses.

#### Determination of the iodine deficiency effect

For continuous data, the mean UIC was transformed into mean differences (MDs) between the groups along with a 95% confidence interval (CI). When mean-adjusted differences (MADs) from change scores were available, we preferred to use them. The MDs and MADs were included in the meta-analysis using the generic inverse variance method. For studies that reported medians and interquartile ranges, the estimated means of the sample and standard deviation were obtained using a method proposed by Hozo and coworkers (13).

For dichotomous outcomes, relative risk (RR) with 95% CI was calculated as an estimate of the effect of UIC of <150 μg/L.

### Assessment of publication biases

When >10 studies were included in the meta-analysis, a funnel plot and Egger’s regression test were used to investigate the presence of publication bias (14).

### Assessment of statistical heterogeneity

Variability in the exposure or intervention effects is known as statistical heterogeneity and is a consequence of clinical or methodological variety, or both, among the studies (14). In this review, the statistical heterogeneity between the results of studies included was ascertained by visual inspection of forest plots (no overlap of CIs around the effect estimates of individual studies), by Higgins or *I*^2^ statistic, in which *I*^2^ >50% indicated a moderate probability of heterogeneity, and using chi-squared tests (Chi2), where *P* < 0.10 indicated heterogeneity (14). If statistical heterogeneity was present among the studies and more than ten studies were included in a meta-analysis, a meta-regression analysis was conducted to investigate potential sources of variability between study characteristics and the evaluated outcomes. Study design (cohort or cross-sectional), mean age of pregnant women, iodine status (sufficient or mild-to-moderate deficiency), gestational trimester (first, second or third) and UIC used as control group (≥150 or 150–249) were used as covariates. The Knapp–Hartung correction was used to calculate the significance of the meta-regression coefficients (15). In order to evaluate whether UICs in one particular gestational trimester differentially associate with the outcomes investigated, when data were available, we performed a subgroup analysis according to the gestational trimester of individual diagnosis of UIC <150 μg/L.

We also incorporate uncertainty in the analyses calculating prediction intervals (PIs), which were derived from the random-effects meta-analysis if Chi2 < 0.10 or *I*^2^ > 35% and more than five studies were included in the analysis. The PI reflects the variation in the exposure or treatment effects over different settings, including the expected effect in future studies (16, 17).

### Sensitivity analysis

Sensitivity analyses were performed by comparing the iodine deficiency effect between published and imputed data, the mean UIC between 150 and 249 vs UIC >150 μg/L, by separating studies that corrected UIC for urinary creatinine from those that did not. Although our analyses are based on individual iodine insufficiency, we also performed a sensitivity analysis according to sample size (dividing the studies that had >500 participants from those that had ≤500 pregnant women). This choice aligns with the findings of Andersen and colleagues, who determined that the number of spot urine samples needed to estimate iodine levels in a population with 95% confidence interval and a precision range of ±5% is approximately 500 (18).

### Quality of evidence

The quality of evidence for estimating the effect of iodine deficiency on outcomes that could be plotted in the meta-analysis was generated following the Grading of Recommendations Assessment, Development and Evaluation (GRADE) Working Group (19). Observational studies had a low certainty of evidence. However, the quality of evidence increased when studies presented with one of the following criteria: large magnitude of the iodine deficiency effect, evidence of a dose–response relationship and decrease in the magnitude of the iodine deficiency effect because of all plausible biases.

## Results

### Selection of studies

**Figure 1:**
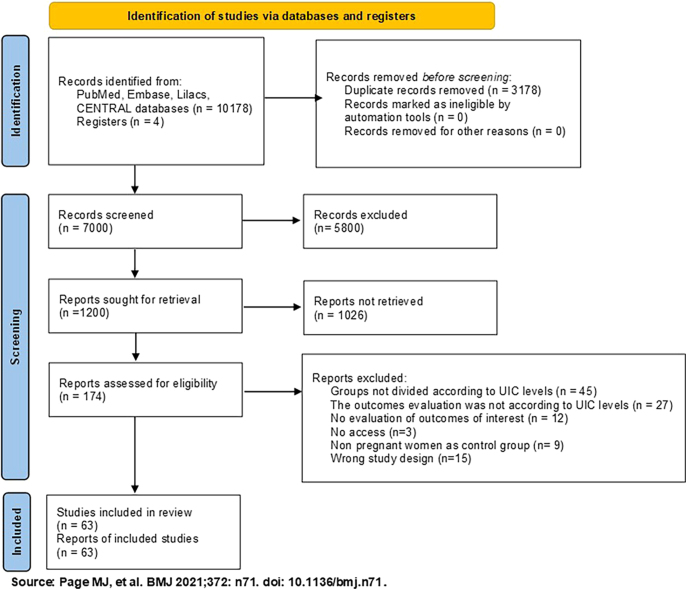
Flow diagram of selected studies.

After removing duplicates, the search strategies yielded a total of 7000 studies. Of these, 174 studies with a high probability of meeting our inclusion criteria were selected (Fig. 1). After fully reading these studies, 63 (comprising a total of 65,636 pregnant women) were included in this review (1, 8, 9, 20, 21, 22, 23, 24, 25, 26, 27, 28, 29, 30, 31, 32, 33, 34, 35, 36, 37, 38, 39, 40, 41, 42, 43, 44, 45, 46, 47, 48, 49, 50, 51, 52, 53, 54, 55, 56, 57, 58, 59, 60, 61, 62, 63, 64, 65, 66, 67, 68, 69, 70, 71, 72, 73, 74, 75, 76, 77, 78, 79) and 111 were excluded because they were not in agreement with the PECO structure. The reasons and references of excluded studies are described in the supplementary data.

### Characteristics of included studies

Regarding the study design, 57% of the included studies were cohort studies and 43% were analytical cross-sectional studies. Their sample size ranged from 35 to 7435 pregnant women. Women in the three gestational trimesters were included. Approximately 48% of the included studies were from Asia, 29% from Europe, 6% from Eurasia, 9% from South America, 3% from Africa and North America and 5% from Australia. All included studies had a group of pregnant women with UIC <150 μg/L. As a control group, 39 studies had UIC between 150 and 249 μg/L and 24 had UIC ≥150 μg/L. Regarding the iodine status, 34, 9 and 15 studies were from regions with sufficient, mildly deficient and mildly to moderately deficient iodine supply, respectively. Other studies did not report on the iodine status. Most of the studies included in our review utilized the UIC from the first trimester to classify pregnant women as iodine sufficient or insufficient and to correlate these classifications with health outcomes. However, a few studies also examined correlations with the second and third trimesters or correlated data only from those later trimesters. Consequently, we preferred to include data primarily from the first trimester because the majority of the studies focused on this time period.

Data regarding the main characteristics of each included study are presented in Table 1.

**Table 1:** Characteristics and summary of findings of 63 included studies that investigated associations of individual UIC in pregnancy with maternal and newborn outcomes.

Study	Pregnant women (*n*)	Mean age of pregnant women (years)	Gestational trimester	Country	Period	Iodine status	Control (UIC μg/L)	Confounders
Abel 2018	2577	30	Second	Norway	1999–2008	Mild to moderate deficiency	≥150	Maternal age, education, parity, prepregnancy BMI and smoking
Aktas 2022	265	28	Third	Turkey	2013	Sufficient	≥150	TPOAb
Alvarez-Pedrerol 2009	557	31	First	Spain	2004–2006	NR	150–249	Parity, mother’s weight and age, gestational age and smoking
Amouzegar 2014	203	25	First	Iran	2004–2006	Sufficient	≥150	NR
Azizi 2011	138	25	First, second, third	Iran	2005–2006	Sufficient	≥150	NR
Bath 2013	958	29	First	England	1991–1992	Mild deficiency	≥150	Maternal age, maternal education, alcohol intake, breastfeeding, fish oil supplements, intake of iron estimated, smoking, birth weight and preterm birth
Berg 2017	197	32	Second	Norway	2007–2009	Mild to moderate deficiency	≥150	Maternal age, education, parity, prepregnancy BMI, smoking, intake of dairy products/marine food/eggs, dietary intake of iodine and gestational age
Charoenratana 2015	399	28	First, second, third	Thailand	2013–2014	NR	≥150	Parity, number of antenatal visits, gestational age, gestational diabetes, pre-eclampsia, postpartum hemorrhage and low Apgar scores
Chen 2018	2078	28	Second, third	China	2014–2016	Sufficient	150–249	Maternal age, alcohol consumption, smoking, average personal income, folic acid supplementation, abortion history, parity, educational level and BMI
Chen 2022	1461	28	First, second, third	China	2016–2018	Sufficient and mild deficiency	150–249	TPOAb
Cho 2015	344	33	First, second, third	Korea	2012–2013	Sufficient	150–249	Age, weight, height, BMI, smoking, parity, region of residence and thyroid autoantibodies
Corcino 2019	243	26	First, third	Brazil	2014–2017	Sufficient	≥150	Age, iodine concentration in table salt, gestational age, BMI, TSH at the time of urine collection, smoking habit, parity and thyroid antibodies
Cui 2022	7435	29	First, second, third	China	2016–2018	Sufficient	150–249	Mother’s age and education
Delshad 2016	1072	27	First, second, third	Iran	2013–2014	Sufficient	150–249	Age, parity and gestational age
Gargari 2020	884	29	Third	Iran	2017–2018	Sufficient	≥150	Gestational age, weight gain during pregnancy, mother’s educational status, smoking and parity
Ghassabian 2014	1525	29	First, second	The Netherlands	2002–2006	Sufficient	≥150	Maternal age and education, child’s ethnicity, breastfeeding, paternal age, maternal BMI, smoking, maternal IQ, marital status, paternal education, maternal folate concentration in early pregnancy and household income
Guo M 2020	500	NR	First, second, third	China	2017–2019	Sufficient	≥150	TPOAb
Guo W 2020	2378	29	First, second, third	China	2016–2017	Sufficient	150–249	TPOAb and TgAb
Gyamfi 2018	239	28	First, second, third	Ghana	January to April 2016	Sufficient	150–249	History of thyroid disease, thyroid medication, thyroidectomy and smoking
Habimana 2014	225	27	First, second, third	Democratic Republic of Congo	2009–2011	Mild deficiency	150–249	Age, parity and socioeconomic area
Hynes 2017	266	29	Second, third	Australia	1999–2001	Mild deficiency	≥150	Gestational age at UI collection, maternal age, gestational length, birth weight and sex and maternal education
Hynes 2013	228	29	Second	Australia	1999–2001	Mild deficiency	≥150	Maternal age, gestational age, gestational length, birth weight, sex, parental education and occupation
Kampouri 2022	1052	26	First	Bangladesh	2002–2003	Deficient	≥150	Age, height, weight, delivery date, education, parity and family socioeconomic status index
Kianpour 2019	418	29	First, second	Iran	NR	Sufficient	≥150	Number of pregnancies, miscarriages and deliveries; infertility; preterm delivery and complications of previous pregnancy; history of autoimmune diseases; diabetes mellitus; and thyroid dysfunction
Koyuncu 2018	440	28	First	Turkey	January to July 2016	Sufficient	150–249	NR
Levie 2019	2009	30	First	Sweden	2007–2010	Mild to moderate deficiency	150–249	Gestational age, maternal BMI, smoking status, child sex and TPOAb
Liu 2022	300	30	First, second, third	China	2021	NR	150–249	NR
Lopes 2023	70	29	Third	Portugal	2010–2021	Mild to moderate deficiency	≥150	Family income and maternal education
Markhus 2018	851	NR	Second	Norway	2011–2014	Mild to moderate deficiency	≥150	Age, prepregnancy weight and height, parity, education, marital status, use of iodine-containing supplements, use of omega-3 supplements in pregnancy and daily smoking in pregnancy
Medici 2013	1098	30	First	The Netherlands	2002–2006	Sufficient	≥150	Maternal age, socioeconomic status and thyroid disease
Mills 2019	329	29	NR	USA	2005–2009	Sufficient	≥150	Woman’s age, woman’s race/ethnicity, woman’s educational level, household income, BMI, diabetes mellitus, consumption of alcohol, smoking and history of thyroid diseases
Mioto 2018	273	28	First, second, third	Brazil	2012–2016	Sufficient	150–249	NR
Morais 2020	214	27	First, third	Brazil	2014–2017	Sufficient	150–249	Maternal age, BMI, smoking, parity, TPOAb and use of LT4 or iodine-containing multivitamins during pregnancy
Moreno-Reyes 2013	1311	28	First, third	Belgium	2010–2011	Mild deficiency	150–249	Sociodemographic and socioeconomic characteristics, smoking and alcohol drinking behavior, thyroid diseases, use of iodine-containing supplements and food consumption, use of iodized household salt and BMI
Murcia 2017	1803	30	First	Spain	2009–2013	Sufficient/mild deficiency	150–249	Maternal educational level, social class, employed at baby’s age 1 year, smoking, history of thyroid disease and age
Murcia 2011	691	30	First	Spain	2005–2007	Mild deficiency	150–249	Educational level, social class, cohabitant, employed at baby’s age 1 year, smoking, history of thyroid disease, infant sex and breastfeeding
Nazarpour 2019	1286	27	First, second, third	Iran	2013–2016	Sufficient	150–249	Age, BMI and TPOAb
Oguz 2012	162	25	Second	Turkey	October to December 2008	Sufficient	≥150	Socioeconomic level, household income, education duration and use of iodized salt
Olivares 2012	77	25	NR	Argentine	March to August 2009	Mild to moderate deficiency	≥150	Arterial hypertension, diabetes, smoking, maternal BMI and smoking
Pan 2019	1099	28	First, second, third	China	2016–2017	Sufficient	150–249	Age, height, weight and trimester
Rajatanavin 2007	1385	NR	NR	Thailand	2000–2003	Mild deficiency	≥150	NR
Rebagliato 2013	1519	NR	First, third	Spain	2003–2008	Mild deficiency	150–249	Maternal age, sex, history of thyroid disease, maternal origin, mixed social class, breastfeeding, parity, educational level
Rebagliato 2010	1844	31	First, second	Spain	2004–2008	Mild deficiency	150–249	Iodine intake, maternal age, country of origin, educational level, prepregnancy BMI, parity and gestational age
Ruiz 2009	154	33	Third	Spain	2006–2007	Mild deficiency	150–249	NR
Ruiz Ochoa 2017	106	31	First	Spain	NR	Sufficient	150–249	Parity, history of miscarriage, iodized salt and pharmacological supplements
Sanowal 2023	400	NR	First, second, third	Eastern Himalayas	2021–2022	Mild to moderate deficiency	150–249	NR
Saraiva 2018	244	26	First	Brazil	2014–2017	Sufficient	150–249	Maternal age, BMI, multiparity, SCH, abnormal iodine concentration in table salt, thyroiditis and smoking habit
Schiller 2020	100	32	First	Israel	NR	Mild deficiency	≥150	Age, gestational age, school education, BMI, parity, smoker, positive family history of thyroid disease and iodine-containing supplement consumption
Shi 2015	7190	NR	First	China	2012–2014	Sufficient	150–249	Age, weeks of gestation, BMI, TPOAb and TgAb
Torlinska 2018	3182	29	First, second, third	United Kingdom	1991–1992	Mild deficiency	150–249	Mother’s BMI (prepregnancy), age, parity, cigarette smoking during early pregnancy and trimester
Ulu 2017	180	26	Third	Turkey	July to September 2011	Mild to moderate deficiency	150–249	Smoking in pregnancy and iodized salt consumption
Vila 2008	35	29	First, third	Spain	First semester of 2000	Mild deficiency	≥150	Iodine supplements and active smokers
Wang 2022	1264	28	First, second, third	China	2019	Sufficient	150–249	TPOAb and TgAb
Wu 2023	562	28	Second	China	2021	Mild deficiency	150–249	Age, gestational age, TPOAb, BMI and parity times
Wu Wen 2023	469	28	First, second, third	China	2016–2019	Sufficient and mild deficiency	150–249	Age, gestational age, maternal and paternal education, parity, smoker and familial income
Xiao 2017	1569	28	First	China	2012–2014	Sufficient	150–249	BMI, abdominal circumference, blood pressure, heart rate, smoking rate, drinking rate, TSH, FT4, TPOAb, TgAb and Tg
Yang 2018	2347	26	Second, third	China	July to September 2015	Sufficient	150–249	Maternal age, education, income and working hours
Yang 2020	2329	NR	First, second, third	China	2015	Sufficient	150–249	TPOAb and TgAb
Yoganathan 2015	477	29	Third	Sri Lanka	NR	NR	150–249	Average intake of iodine-rich food by the mothers, age, height, weight and educational attainment
Zha 2023	212	30	The second half of pregnancy	China	2018–2021	Mild to moderate deficiency	150–249	Age, gestational age, TPOAb, BMI and parity times
Zhang 2018	988	28	First, second, third	China	2015–2017	Sufficient	150–249	Maternal age, BMI, gestation duration, mode of delivery, parity, maternal TSH, maternal UIC, neonatal sex, neonatal weight and height and trimester when mother joined the study
Zhang 2021	726	30	First	China	2017–2019	Sufficient	150–249	Age, BMI, birth order and history of spontaneous abortion
Zhou 2018	699	30	First, second	Australia	2011–2015	Mild deficiency	150–249	Mother’s age, education, employment status, number of adults and children living in the household, maternal BMI, smoking, gestational age, parity and breastfeeding at discharge from hospital after birth

Abbreviations: UIC, urinary iodine concentration; NR, not related; BMI, body mass index; TPOAb, thyroid peroxidase antibodies; FT4, free thyroxine; Tg, thyroglobulin; TSH, thyroid-stimulating hormone; IQ, intelligence quotient; SCH, subclinical hypothyroidism.

### Risk of bias of the included studies

Supplementary Tables S1 and S2 (see section on Supplementary materials given at the end of the article) show the risk of bias in the included studies. This assessment had the same classification for all outcomes. For the majority of the domains, the questions were responded as yes.

### Meta-analysis

Evaluation of the included studies revealed that most outcomes analyzed were dichotomous, except maternal TSH level, thyroid volume and birth length, which were continuous data. Data from 49 studies were included in the meta-analyses. Details of each outcome are described below.

### Maternal outcome

**Figure 2:**
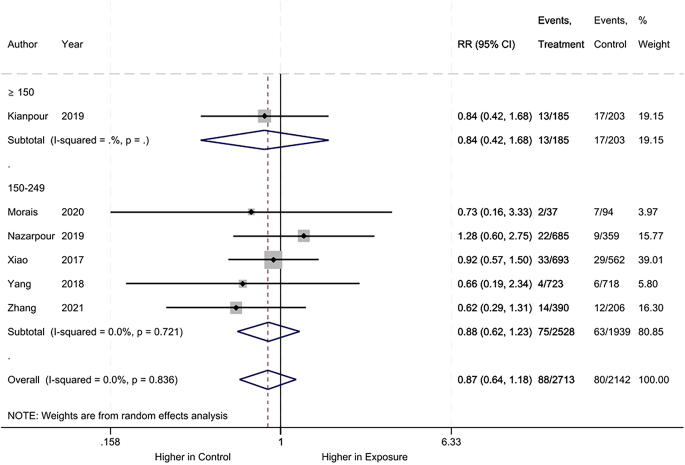
Meta-analysis of miscarriage. Subgroup analysis based on UICs used as control (UIC between 150 and 249 μg/L vs UIC ≥150 μg/L).

For miscarriage, we found no difference between the iodine deficiency and control groups (RR: 0.87, 95% CI: 0.64–1.18, 6 studies, 4855 participants, *I*^2^ = 0, Fig. 2; low certainty of evidence (Table 2)).

**Table 2:** Summary of findings: quality of evidence based on the GRADE approach of the association of individual UICs in pregnancy with maternal and newborn outcomes.

Outcomes	Anticipated absolute effects* (95% CI)		Relative effect (95% CI)	No. of participants (studies)	Certainty of the evidence (GRADE)	Comments
	Risk with (comparison)	Risk with (exposure)				
Preterm birth	57 per 1000	**69 per 1000** (55–85)	**RR 1.20** (0.97–1.48)	15,644 (13 observational studies)	⨁⨁◯◯ Low	The evidence is very unclear about the effect of ioduria in pregnancy with <150 μg/L on preterm birth
Low birth weight	34 per 1000	**42 per 1000** (30–60)	**RR 1.25** (0.88–1.78)	10,775 (10 observational studies)	⨁⨁◯◯ Low	The evidence is very unclear about the effect of ioduria in pregnancy with <150 μg/L on low birth weight
Stillbirth	8 per 1000	**7 per 1000** (3–15)	**RR 0.79** (0.34–1.82)	3406 (5 observational studies)	⨁◯◯◯ Very low^a^	The evidence is very unclear about the effect of ioduria in pregnancy with <150 μg/L on stillbirth
Miscarriage	37 per 1000	**32 per 1000** (24–44)	**RR 0.87** (0.64–1.18)	4855 (6 observational studies)	⨁⨁◯◯ Low	Ioduria in pregnancy with <150 may not increase abortion
Maternal subclinical hypothyroidism	43 per 1000	**42 per 1000** (33–54)	**RR 0.98** (0.77–1.25)	15,117 (16 observational studies)	⨁⨁◯◯ Low	The evidence is unclear about the association of low UICs with maternal subclinical hypothyroidism
Maternal TSH measurement	The mean TSH measurement was **0** UI/mL	MD **0.02 UI/mL higher** (0.02 lower to 0.06 higher) ccc	-	21,531 (26 observational studies)	⨁⨁◯◯ Low	Ioduria in pregnancy with <150 μg/L may result in no difference in maternal TSH measurement

**Population**: pregnant women. **Exposure**: iodine deficiency; we defined iodine deficiency as a median UIC of <150 μg/L. **Comparison**: iodine sufficiency; we defined iodine sufficiency as a median UIC between 150 and 249 μg/L. GRADE Working Group grades of evidence. **High certainty**: we are very confident that the true effect lies close to the effect estimate. **Moderate certainty**: we are moderately confident in the effect estimate. The true effect is likely to be close to the estimate of the effect; however, there is a possibility that it is substantially different. **Low certainty**: our confidence in the effect estimate is limited. The true effect may be substantially different from the estimate of the effect. **Very low certainty**: we have very little confidence in the effect estimate. The true effect is likely to be substantially different from the estimate of effect. a, low limit of confidence interval under 0.75.

* **The risk in the intervention group** (and its 95% confidence interval) is based on the assumed risk in the comparison group and the **relative effect** of the intervention (and its 95% CI). **CI**, confidence interval; **MD**, mean difference; **OR**, odds ratio; **RR**, risk ratio.

**Figure 3:**
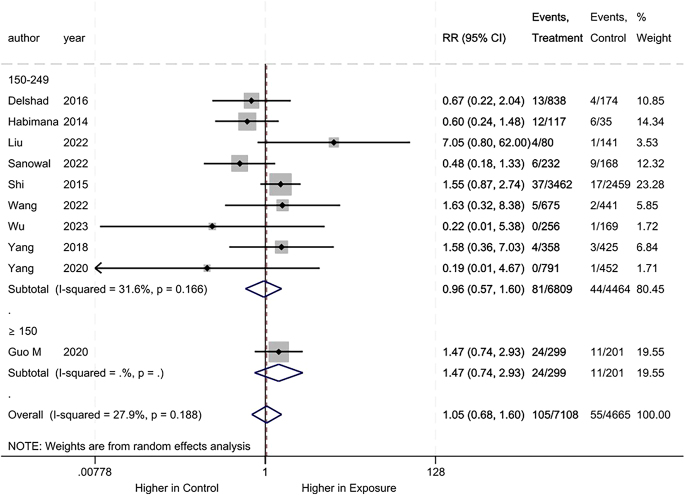
Meta-analysis of maternal hypothyroidism. Subgroup analysis based on UICs used as control (UIC between 150 and 249 μg/L vs UIC ≥150 μg/L).

Regarding maternal hypothyroidism, we found no difference between the iodine deficiency and control groups (RR: 1.05, 95% CI: 0.68–1.60, 10 studies, 11,773 participants, *I*^2^ = 27.9%, Fig. 3).

**Figure 4:**
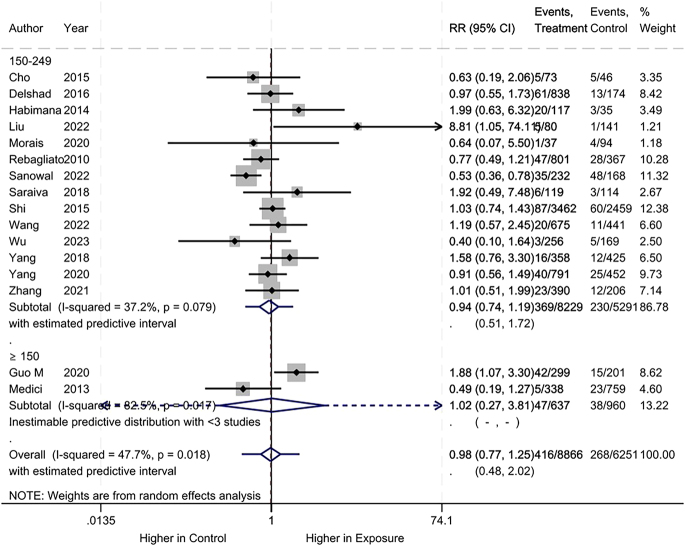
Meta-analysis of maternal subclinical hypothyroidism. Subgroup analysis based on UICs used as control (UIC between 150 and 249 μg/L vs UIC ≥150 μg/L).

Regarding maternal subclinical hypothyroidism, we found no difference between the iodine deficiency and control groups (RR: 0.98, 95% CI: 0.77–1.25, 16 studies, 15,117 participants, *I*^2^ = 7.7%, Fig. 4; low certainty of evidence (Table 2)).

For thyroid volume, no difference was observed between the iodine deficiency and control groups (MD: −0.50, 95% CI: −1.02 to 0.02, 4 studies, 620 participants, *I*^2^ = 0, supplementary data).

**Figure 5:**
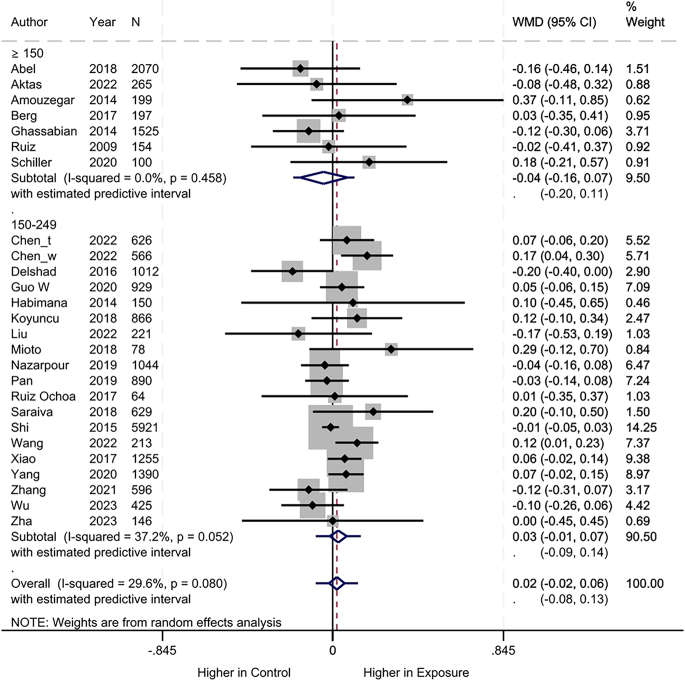
Meta-analysis of maternal TSH measurement. Subgroup analysis based on UICs used as control (UIC between 150 and 249 μg/L vs UIC ≥150 μg/L).

Regarding maternal TSH measurement, no difference was found between the iodine deficiency and control groups (MD: 0.02, 95% CI: −0.02 to 0.06, 26 studies, 21,531 participants, *I*^2^ = 37.2%, Fig. 5; low certainty of evidence (Table 2)).

### Neonatal outcomes

**Figure 6:**
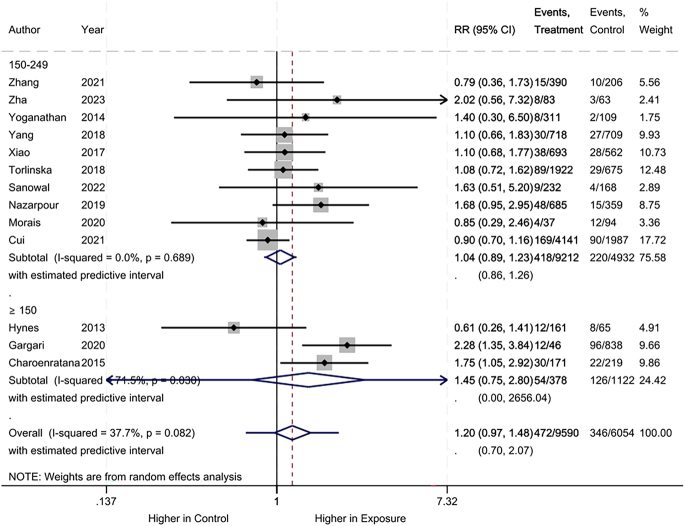
Meta-analysis of preterm birth. Subgroup analysis based on UICs used as control (UIC between 150 and 249 μg/L vs UIC ≥150 μg/L).

Regarding preterm birth, no difference was observed between the iodine deficiency and control groups (RR: 1.20, 95% CI: 0.97–1.48, 13 studies, 15,644 participants, *I*^2^ = 37.7%, Fig. 6; low certainty of evidence (Table 2)). No difference was maintained when we separated the groups based on UICs used as control (UIC between 150 and 249 μg/L vs UIC >150 μg/L) based on the population size (>500 vs ≤500) and correcting UIC for urinary creatinine (supplementary data).

**Figure 7:**
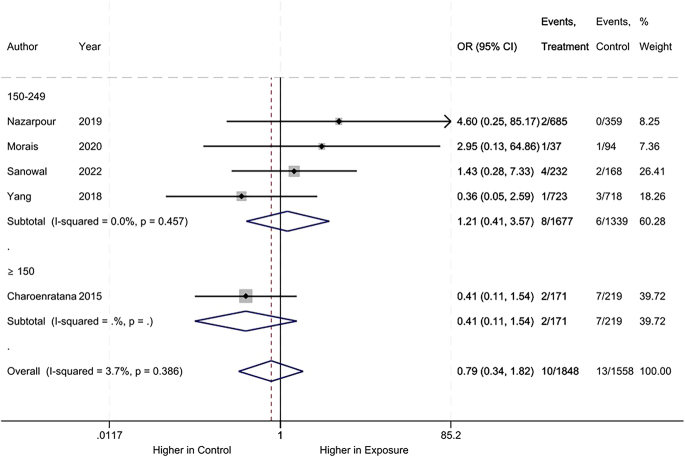
Meta-analysis of stillbirth. Subgroup analysis based on UICs used as control (UIC between 150 and 249 μg/L vs UIC ≥150 μg/L).

Regarding stillbirth, no difference was observed between the iodine deficiency and control groups (RR: 0.79, 95% CI: 0.34–1.82, 5 studies, 3406 participants, *I*^2^ = 3.7%, Fig. 7; very low certainty of evidence (Table 2)).

**Figure 8:**
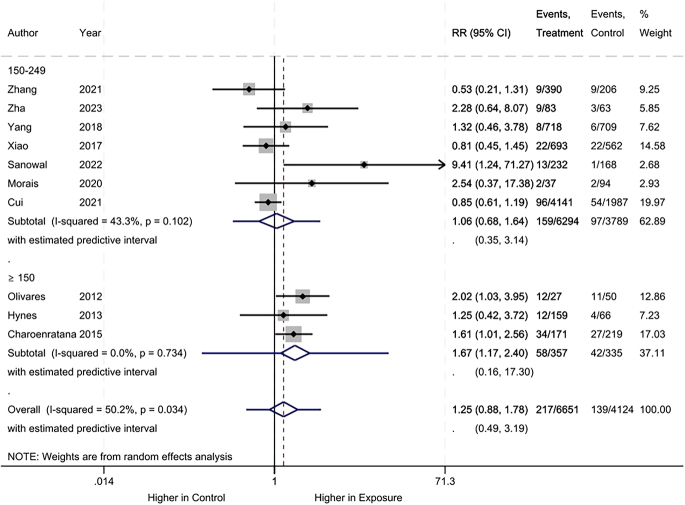
Meta-analysis of low birth weight. Subgroup analysis according to UICs used as control (UIC between 150 and 249 μg/L vs UIC ≥150 μg/L).

Regarding low birth weight, no difference was observed between the iodine deficiency and control groups (RR: 1.25, 95% CI: 0.88–1.78, 10 studies, 10,775 participants, *I*^2^ = 50.2%, Fig. 8; low certainty of evidence (Table 2)).

**Figure 9:**
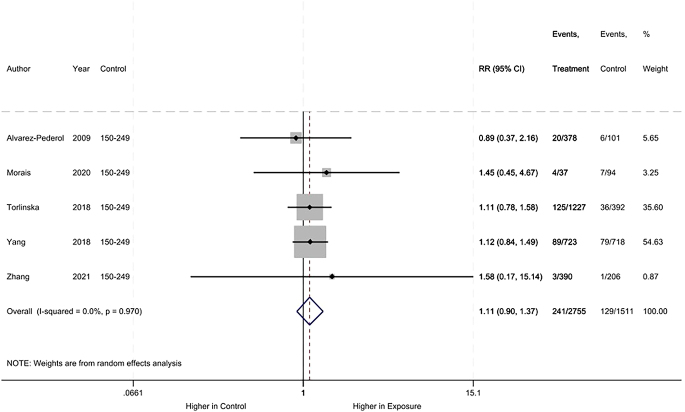
Meta-analysis of small for gestational age.

Regarding small for gestational age, no difference was observed between the iodine deficiency and control groups (RR: 1.11, 95% CI: 0.90–1.37, 5 studies, 4266 participants, *I*^2^ = 0, Fig. 9).

**Figure 10:**
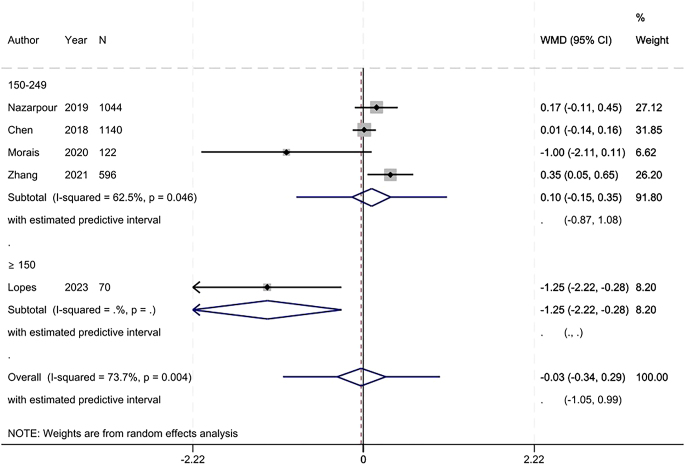
Meta-analysis of birth length. Subgroup analysis based on UICs used as control (UIC between 150 and 249 μg/L vs UIC ≥150 μg/L).

For birth length, no difference was observed between the iodine deficiency and control groups (MD: −0.03 cm, 95% CI: −0.34 to 0.29, 5 studies, 2972 participants, *I*^2^ = 73.7%, Fig. 10).

Regarding elevated neonatal TSH, no difference was observed between the iodine deficiency and control groups (RR: 1.29, 95% CI: 0.77–2.18, 3 studies, 2030 participants, *I*^2^ = 52.2%, supplementary data).

In all analyses, the direction of the exposure effect was maintained when sensitivity analyses were performed by separating the groups based on population size (>500 vs ≤500), according to the gestational trimester and maternal TSH, and by correcting UIC for urinary creatinine (supplementary data).

We investigated statistical heterogeneity using meta-regression for the frequency of prematurity, low birth weight, maternal hypothyroidism, maternal subclinical hypothyroidism and maternal TSH levels. However, the individual and joint tests for all covariates (study design, mean age of pregnant women, iodine status, gestational trimester and UIC used as control group) gave a *P* value >0.05, indicating no evidence of association between them and the size of the exposure effect (supplementary data). The subgroup analysis of UIC used as control group (UIC ≥150 vs 150–249 μg/L) also did not change the direction of the effect size (supplementary data).

The quality of evidence for the main outcomes is presented in Table 2. None of the results met the criteria that would support an increase in evidence quality (80). For the outcome of maternal TSH measurement, the certainty was rated down one level due to the lower limit of the confidence interval being below 0.75.

### Outcomes not reported in the quantitative synthesis

Eleven studies evaluated the association between UIC levels in pregnant women and scores of adverse infant neuromotor development/IQ (8, 21, 27, 35, 41, 45, 50, 56, 73, 75, 78). However, a meta-analysis of these findings cannot be performed because the studies used different tools with distinct domains for evaluating this association.

The findings of these 11 studies were different from one another, and only some studies had reported on the association between UIC <150 μg/L and scores of adverse infant neuromotor development/IQ. Bath and coworkers (27) evaluated the association between maternal iodine status and child’s IQ at the age of 8 years and reading ability at the age of 9 years. They observed that children of women with an iodine-to-creatinine ratio <150 μg/g were more likely to have scores in the lowest quartile for verbal IQ at the age of 8 years (odds ratio (OR): 1.58, 95% CI: 1.09–2.30, *n* = 880; *P* = 0.02) than those of women with a ratio ≥150 μg/g. A similar trend was observed for reading accuracy (OR: 1.69, 95% CI: 1.15–2.49, *n* = 839; *P* = 0.007) and reading comprehension (OR: 1.54, 95% CI: 1.06–2.23, *n* = 839; *P* = 0.02) at the age of 9 years. Murcia and coworkers (78) assessed children using the McCarthy Scales of Children’s Abilities up to the age of 4–5 years and found an association between low UIC and lower cognitive scores. After adjusting for creatinine, children of women with UIC/Cr <100 μg/L had a general cognitive score of −3.93 (95% CI −6.18 to −1.69), which were lower than those of women with UIC/Cr in the range of 150–249 μg/L. Kampouri and coworkers (41) analyzed cognitive abilities assessed using the Wechsler Preschool and Primary Scale of Intelligence and the Wechsler Intelligence Scale for Children in children aged 5 and 10 years. In their study, maternal UIC <150 μg/L was associated with lower full-scale scores at the age of 5 and 10 years, particularly verbal scales, compared with those of the reference groups. Similarly, Hynes and coworkers (8) compared educational outcomes of children aged 9 years, whose mothers had UIC <150 μg/L or >150 μg/L during pregnancy. They concluded that children whose mothers had UIC <150 μg/L showed reductions of 10.0% in spelling (−41.1 points, 95% CI: −68.0 to −14.3, *P* = 0.003), 7.6% in grammar (−30.9 points, 95% CI: −60.2 to −1.7, *P* = 0.038) and 5.7% in English literacy (−0.33 points, 95% CI −0.63 to −0.03, *P* = 0.034) performance compared with children whose mothers’ UICs were >150 μg/L.

Conversely, Rebagliato and coworkers (56) assessed neuropsychological development using the Bayley Scales of Infant Development in infants of median age 16 months. Combined adjusted analyses revealed no difference in infants from mothers with UICs of 150–249 μg/L and those from mothers with UIC <100 μg/L. Markhus and coworkers (45) explored the association between maternal UIC and neurodevelopment in children aged 6, 12 and 18 months using the Bayley Scales of Infant and Toddler Development. Although UIC <100 μg/L was associated with reduced receptive (*P* = 0.025) and expressive language skills (*P* = 0.002), no reduced cognitive or fine and gross motor skills were observed. In addition, the median UIC in pregnancy was 78 μg/L (interquartile range (IQR): 46 and 130), which is classified as insufficient iodine intake according to the WHO. Murcia and coworkers (50) administered the Psychomotor Development Index using the Bayley Scales of Infant Development in children aged 1 year, and multivariate linear and logistic analyses revealed no associations between the Mental Development Index and UIC levels. Zhou and coworkers (73) assessed childhood neurodevelopment using the Bayley Scales of Infant and Toddler Development (Bayley-III) in children aged 18 months and found no association between UIC in pregnancy and Bayley-III outcomes. Similar results were observed by Ghassabian and coworkers (35), who evaluated nonverbal IQ and language comprehension of a 6-year-old children and concluded that UIC <150 μg/g creatinine was not associated with children’s nonverbal IQ (adjusted OR: 1.33, 95% CI: 0.92–1.93), and there was no relation between maternal UIC and children’s language comprehension at 6 years of age.

Wu and coworkers (75) categorized maternal UIC into four groups: <100 μg/L (moderate deficiency), 100–149 μg/L (mild deficiency), 150–249 μg/L (sufficiency) and ≥250 μg/L. After multivariable adjustment, they found that infants born to mothers with UIC ≥250 μg/L had an increased risk of adaptive developmental delay (OR: 2.38; 95% CI: 1.06–5.35) compared with infants born to mothers with UIC 150–249 μg/L. Furthermore, infants born to mothers with mild iodine deficiency showed a 4.13-point improvement in their personal–social domain developmental quotient $\left(P\;\frac{1}{4}\;0.046\right)$ compared with infants born to mothers with 150–249 μg/L UIC during pregnancy.

Lopes and coworkers did not find any association between UIC levels during pregnancy on the long-term growth and neurodevelopment of children (21).

No included studies evaluated neonatal goiter, neonatal thyroid volume and death in the first year of life. Congenital malformations were only assessed by a single study, and no differences were found between the UIC groups (32).

### Publication bias

Publication bias was investigated for preterm birth, and in the funnel plot, asymmetries were not observed. Thus, the Egger test was performed, with a *P* value >0.05 (supplementary data).

## Discussion

The present study evaluated the association of individual UIC (iodine deficiency: UIC <150 μg/L and control: UIC: 150–249 μg/L) in pregnancy with maternal and newborn outcomes. The outcomes plotted in meta-analyses were spontaneous miscarriage, hypothyroidism, subclinical hypothyroidism, maternal thyroid volume, maternal TSH measurement, preterm birth, stillbirth, low birth weight, small for gestational age, neonatal elevated TSH and birth length.

No association was observed between UIC <150 μg/L during pregnancy and maternal and fetal outcomes. However, upon individually analyzing the included studies, some of them showed an iodine deficiency effect on maternal or fetal outcomes; however, some confounders explained the differences between groups. Charoenratana and coworkers (9) reported the association between UIC <150 μg/L and increased risk of preterm birth and low birth weight based on the higher prevalence of UIC <150 μg/L than expected for the population evaluated and insufficiency of other nutritional factors in pregnant women. Gargari and coworkers (34) also observed an increase in preterm birth in the group with UIC <150 μg/L; they identified weight gain during pregnancy, interval between the most recent pregnancies, unplanned pregnancy and the absence of nutritional complement consumption as probable confounders.

Regarding neurodevelopmental outcomes, in the current review, studies conducted on children older than 4–5 years demonstrated some association between neuromotor development/IQ and UIC levels, but studies evaluating younger children have not consistently shown this association. We propose that this discrepancy is primarily attributable to the inherent limitations of the included studies than any true association between these two variables. In addition, a review by Monaghan and coworkers (81) reported findings similar to those of our review, and the lack of association in these studies could be explained by the different types of cognitive testing conducted and the children selected.

During the performance of this review, two systematic reviews were published on the same subject. Nazeri and coworkers (82) analyzed the mean birth weight, length and head circumference of newborns whose mothers had UIC <150 or >150 μg/L during pregnancy and concluded that these anthropometric measures were not associated with maternal UIC during pregnancy. Nazarpour and coworkers (83) revealed no evidence of an association between the odds of preterm birth, low birth weight and hypertensive disorders of pregnancy in euthyroid pregnant women between UIC groups. Therefore, these results emphasize that UIC <150 μg/L is not related to maternal and neonatal outcomes. Our review included more studies and consequently more participants than the two aforementioned published reviews: Nazeri and coworkers (82) included 19 studies in the review and 11 in the meta-analyses, whereas Nazarpour and coworkers (83) included only six studies. A total of 63 studies were included in our systematic review and 49 in the meta-analyses, with a total of 65,636 pregnant women assessed. In addition, sensitivity analyses were performed by separating studies according to the population size of >500 or by correcting UIC for urinary creatinine and graduated the quality of evidence based on the GRADE approach. Another systematic review was conducted regarding maternal iodine status and birth outcomes; however, the authors investigated potential threshold effects of UIC on these outcomes, rather than directly comparing the effect of UIC levels below 150 μg/L with those between 150 and 249 μg/L (84). They included 24 cohort studies encompassing a total of 42,503 participants. However, nine of these studies were excluded from our review as they did not evaluate the association between low UIC (UIC <150 vs 150–249) and adverse maternal and neonatal outcomes (84, 85, 86, 87, 88, 89, 90, 91, 92).

Nevertheless, our review has some limitations. The main limitation is regarding the design of the included studies; all of them are observational studies. Thus, we could not evaluate the influence of the known and unknown confounders in the analyses. Another limitation is associated with the eligibility criteria; we selected patients with UICs between 150 and 249 μg/L for comparison; however, some studies classified normal UICs as being ≥150 μg/L. Another limitation was that the ages of children at neurological, motor or IQ assessments differed. Confounders of each study, such as maternal BMI, age, education, occupation, ethnicity, parity, socioeconomic status and antibodies, are other limitations, which may be sources of heterogeneity observed across the included studies. Although we specified that studies involving populations with severe iodine deficiency would be excluded, it is important to note that none of the 111 studies that were excluded from our review were eliminated based on the iodine status of their populations (as detailed in the supplementary data). In the included studies, the iodine status of the population was classified by the authors as being sufficient, mildly deficient and mildly to moderately deficient iodine supply.

Regarding the UIC measurement, Andersen and coworkers considered that the average value of spot UIC is a reliable measure of iodine intake in populations of at least 500 individuals (18). In our systematic review, we included studies with a sample size of <500 participants, and to analyze differences between the groups in robust data, we performed a sensitivity analysis, separating studies with >500 women from those with a smaller sample.

Despite these limitations, our review provides data regarding the association between individual diagnosis of UIC levels during pregnancy and maternal and infant outcomes. In addition, discussing the population median UIC as an indicator of populational diagnosis of iodine deficiency was out of the scope of this review. Our main question was understanding whether individual diagnosis of UIC <150 μg/L in pregnant woman was associated with maternal and newborn adverse outcomes.

In conclusion, no association was observed between individual diagnosis of UIC <150 μg/L in pregnant women and maternal and newborn outcomes, including spontaneous miscarriage, hypothyroidism, subclinical hypothyroidism, maternal thyroid volume, mean TSH, preterm birth, stillbirth, low birth weight, small for gestational age, neonatal elevated TSH and birth length. These findings emphasize the WHO recommendation that medium UIC has been the best indicator to use in population surveys to assess the iodine nutrition of pregnant and lactating women, and it should not be used for the purposes of individual diagnosis and treatment (7). Further research is needed to determine the association between urinary iodine levels during pregnancy and scores of adverse infant neuromotor development/suboptimal IQ.

## Supplementary materials



## Declaration of interest

The authors declare that the work was conducted in the absence of any commercial or financial relationships that could be construed as a potential conflict of interest.

## Funding

This work was supported by the São Paulo Research Foundation (FAPESP)https://doi.org/10.13039/501100001807 (Grant No. 2020/09621-1).

## Author contribution statement

VSNN, GMFSM, CRN and FB conceptualized and designed the study. VSNN and FB developed the search strategies. FB and MBM independently screened eligible studies and extracted data from included studies. FB, MBM and SESF assessed the individual risk of bias. VSNN and FB performed the meta-analyses. VSNN supervised all the phases of this review and refereed any disagreement to avoid errors. All authors participated in data synthesis and assessment of the quality of evidence. All authors critically revised the manuscript and approved its final version.

## Data availability

All data generated or analyzed for this systematic review are included in this published article and its supplementary data.
