# Supplementary material for: Association between individual urinary iodine concentrations in pregnant women and maternal/newborn outcomes
Source: Endocr Connect. 2025 Jan 29;14(3):e240621. doi: 10.1530/EC-24-0621 (PMC11799753; doi:10.1530/EC-24-0621)
Supplement: Supplementary file 1 [file supplementary_materials.pdf]

Association between individual urinary iodine concentrations in pregnant women and maternal/newborn outcomes: A systematic review and meta-analysis.

Fernanda Bolfi[1], Maryan Borcsik Marum[1], Samantha Ellen da Silva Fonseca[1], Glaucia Maria Ferreira da Silva Mazeto[1], Celia Regina Nogueira de Camargo[1], Vania dos Santos Nunes-Nogueira[1]

Supplementary Data

[1]Department of Internal Medicine, São Paulo State University (UNESP) , Medical School, Botucatu, Sao Paulo, Brazil

*** Correspondence:**

Vania dos Santos Nunes Nogueira

E-mail: vania.nunes-nogueira@unesp.br

Keywords: Iodine, Urine, Pregnancy, Systematic Review, Meta-analysis

S1. Table. Risk of bias assessed according to the JBI critical appraisal checklist for cohort studies……………………………………………………………………………page 4

S2. Table. Risk of bias assessed according to the JBI critical appraisal checklist for cross-sectional studies…………………………………………………………………page 5

S1 Fig. Meta-analysis of miscarriage according to the number of participants in the study………………………………………………………………………………..page 7

S2 Fig. Meta-analysis of miscarriage according to the studies that corrected UIC for urinary creatinine……………………………………………………………………..page 7

S3 Fig. Meta-analysis of maternal subclinical hypothyroidism according to the number of participants in the study………………………………………………………page 8

S4 Fig. Meta-analysis of maternal subclinical hypothyroidism according to the studies that corrected UIC for urinary creatinine…………………………………………..page 9

S5 Fig. Meta-analysis of thyroid volume………………………………………...page 10

S6 Fig. Meta-analysis of maternal TSH measurement by comparing the exposure effect according to published vs. imputed data…………………………………………page 11

S7 Fig. Meta-analysis of maternal TSH measurement by comparing the exposure effect according to the number of participants in the study……………………………..page 12

S8 Fig. Meta-analysis of maternal TSH measurement by comparing the exposure effect according to the studies that corrected UIC for urinary creatinine………………page 13

S9 Fig. Meta-analysis of maternal TSH measurement by comparing the exposure effect according to the gestational trimester……………………………………………page 14

S10 Fig. Meta-analysis of preterm birth, by comparing the exposure effect according to the number of participants in the study…………………………………………..page 15

S11 Fig. Meta-analysis of preterm birth by comparing the exposure effect according to the studies that corrected UIC for urinary creatinine……………………………page 16

S12 Fig. Meta-analysis of low birthweight by comparing the exposure effect according to the number of participants in the study………………………………………..page 17

S13 Fig. Meta-analysis of low birthweight by comparing the exposure effect according to the studies that corrected UIC for urinary creatinine…………………………page 18

S14 Fig. Meta-analysis of elevated neonatal TSH………………………………page 19

S15 Fig: Funnel plot of preterm birth……………………………………………page 19

S16 Fig. Egger test of preterm birth……………………………………………page 20
S17 Fig. Meta-regression analysis of frequency prematurity for the covariates study design, iodine status and UIC used as control group……………………………..page 20
S18 Fig. Meta-regression analysis of frequency prematurity for the covariate age……………………………………………………………………………..page 20
S19 Fig. Meta-regression analysis of frequency of low birthweight for the covariates study design, iodine status and UIC used as control group………………………page 21
S20 Fig. Meta-regression analysis of frequency of low birthweight for the covariate age……………………………………………………………………………..page 21
S21 Fig. Meta-regression analysis of frequency of maternal hypothyroidism for the covariates study design, iodine status and UIC used as control group…………...page 22
S22 Fig. Meta-regression analysis of frequency of maternal hypothyroidism for the covariate age………………………………………………………………...page 22
S23 Fig. Meta-regression analysis of frequency of maternal subclinical hypothyroidism for the covariates study design, iodine status and UIC used as control group……page 23
S24 Fig. Meta-regression analysis of frequency of maternal subclinical hypothyroidism for the covariate age………………………………………………………page 23
S25 Fig. Meta-regression analysis of frequency of maternal TSH for the covariates study design, iodine status and UIC used as control group……………………………..page 23
S26 Fig. Meta-regression analysis of frequency of maternal TSH for the covariate age
Search strategies……………………………………………………… …....page 24
References of the excluded studies……………………………………………page 26
Reasons of the excluded studies……………………………………………….page 35

S1. Table. Risk of bias assessed according to the JBI critical appraisal checklist for cohort studies

		Critical appraisal checklist for cohort studies											
**AUTHOR**	**YEAR**	**Q1**	**Q2**	**Q3**	**Q4**	**Q5**	**Q6**	**Q7**	**Q8**	**Q9**	**Q10**	**Q11**	**Include?**
Berg	2017	Y	Y	Y	Y	N	Y	Y	Y	Y	NA	Y	Y
Chen	2018	Y	Y	Y	Y	Y	Y	Y	Y	Y	NA	Y	Y
Hynes	2017	Y	Y	Y	Y	Y	Y	Y	Y	N	Y	Y	Y
Kianpour	2019	Y	Y	Y	Y	Y	Y	Y	Y	Y	NA	Y	Y
Levie	2019	Y	Y	Y	Y	Y	Y	Y	Y	Y	NA	Y	Y
Markhus	2018	Y	Y	Y	Y	Y	Y	Y	Y	Y	NA	Y	Y
Murcia	2017	Y	Y	Y	Y	Y	Y	Y	Y	Y	NA	Y	Y
Nazarpour	2019	Y	Y	Y	N	NA	Y	Y	Y	Y	NA	Y	Y
Ruiz Ochoa	2017	Y	Y	Y	Y	Y	Y	Y	Y	Y	NA	Y	Y
Torlinska	2018	Y	Y	Y	Y	Y	Y	Y	Y	Y	NA	Y	Y
Xiao	2017	Y	Y	Y	Y	Y	Y	Y	Y	Y	NA	Y	Y
Yang	2018	Y	Y	Y	Y	Y	Y	Y	Y	Y	NA	Y	Y
Zhang	2018	Y	Y	Y	Y	Y	Y	Y	Y	Y	NA	Y	Y
Zhou	2018	Y	Y	Y	Y	Y	Y	Y	Y	Y	NA	Y	Y
Alvarez-Pedrerol	2009	Y	Y	Y	Y	Y	Y	Y	Y	Y	NA	Y	Y
Amouzegar	2014	Y	Y	Y	N	NA	Y	Y	Y	Y	NA	Y	Y
Bath	2013	Y	Y	Y	Y	Y	Y	Y	Y	Y	NA	Y	Y
Charoenratana	2015	Y	Y	Y	Y	Y	Y	Y	Y	Y	NA	Y	Y
Corcino	2019	Y	Y	Y	Y	Y	Y	Y	Y	N	N	Y	Y
Ghassabian	2014	Y	Y	Y	Y	Y	Y	Y	Y	Y	NA	Y	Y
Hynes	2013	Y	Y	Y	Y	Y	Y	Y	Y	Y	NA	Y	Y
Mills	2019	Y	Y	Y	Y	Y	Y	Y	Y	N	Y	Y	Y
Medici	2013	Y	Y	Y	Y	Y	Y	Y	Y	Y	NA	Y	Y
Murcia	2011	Y	Y	Y	Y	Y	Y	Y	Y	Y	NA	Y	Y
Olivares	2012	Y	Y	Y	Y	Y	Y	Y	Y	Y	NA	Y	Y
Rebagliato	2013	Y	Y	Y	Y	Y	Y	Y	Y	Y	NA	Y	Y
Vila	2008	Y	Y	Y	Y	N	Y	Y	Y	Y	NA	Y	Y
Yoganathan	2015	Y	Y	Y	N	NA	Y	Y	Y	Y	NA	Y	Y
Zhang	2021	Y	Y	Y	Y	Y	Y	Y	Y	Y	NA	Y	Y
Morais	2020	Y	Y	Y	Y	Y	Y	Y	Y	Y	NA	Y	Y

Kampouri	2022	Y	Y	Y	Y	Y	Y	Y	Y	Y	NA	Y	Y
Cui	2021	Y	Y	Y	Y	Y	Y	Y	Y	Y	NA	Y	Y
Aktas	2022	Y	Y	Y	NA	Y	Y	Y	Y	Y	Y	Y	Y
Zha	2023	Y	Y	Y	Y	NA	Y	Y	Y	Y	N	Y	Y
Liu	2022	Y	Y	Y	NA	NA	Y	Y	Y	Y	Y	Y	Y
Wu Wen	2023	Y	Y	Y	Y	Y	Y	Y	Y	Y	Y	Y	Y

Y: Yes N: No NA: Not Available

**Q1**	Were the two groups similar and recruited from the same population?
**Q2**	Were the exposures measured similarly to assign people to both exposed and unexposed groups?
**Q3**	Was the exposure measured in a valid and reliable way?
**Q4**	Were confounding factors identified?
**Q5**	Were strategies to deal with confounding factors stated?
**Q6**	Were the groups/participants free of the outcome at the start of the study (or at the moment of exposure)?
**Q7**	Were the outcomes measured in a valid and reliable way?
**Q8**	Was the follow up time reported and sufficient to be long enough for outcomes to occur?
**Q9**	Was follow up complete, and if not, were the reasons to loss to follow up described and explored?
**Q10**	Were strategies to address incomplete follow up utilized?
**Q11**	Was appropriate statistical analysis used?

S2. Table. Risk of bias assessed according to the JBI critical appraisal checklist for cross-sectional studies

		**Critical appraisal checklist for analytical cross-sectional studies**								
**AUTHOR**	**YEAR**	**Q1**	**Q2**	**Q3**	**Q4**	**Q5**	**Q6**	**Q7**	**Q8**	**Include?**
Abel	2018	Y	Y	Y	Y	Y	Y	Y	Y	Y
Gyamfi	2018	Y	Y	Y	Y	Y	N	Y	Y	Y
Koyuncu	2018	Y	Y	Y	Y	N	N	Y	Y	Y
Mioto	2018	Y	Y	Y	Y	N	N	Y	Y	Y
Pan	2019	Y	Y	Y	Y	Y	N	Y	Y	Y
Saraiva	2018	Y	Y	Y	Y	Y	Y	Y	Y	Y
Ulu	2017	Y	Y	Y	Y	Y	N	Y	Y	Y
Azizi	2011	Y	Y	Y	Y	N	N	Y	Y	Y
Delshad	2016	Y	Y	Y	Y	Y	N	Y	Y	Y
Habimana	2014	Y	Y	Y	Y	Y	N	Y	Y	Y
Moreno-Reyes	2013	Y	Y	Y	Y	Y	N	Y	Y	Y

Oguz	2012	Y	Y	Y	Y	Y	N	Y	Y	Y
Rajatanavin	2007	Y	Y	Y	Y	N	N	Y	Y	Y
Rebagliato	2010	Y	Y	Y	Y	Y	Y	Y	Y	Y
Ruiz	2009	Y	Y	Y	Y	N	N	Y	Y	Y
Shi	2015	Y	Y	Y	Y	Y	Y	Y	Y	Y
Cho	2015	Y	Y	Y	Y	Y	N	Y	Y	Y
Gargari	2020	Y	Y	Y	Y	Y	Y	Y	Y	Y
Schiller	2020	Y	Y	Y	Y	Y	Y	Y	Y	Y
Yang	2020	Y	Y	Y	Y	Y	Y	Y	Y	Y
Guo W	2020	Y	Y	Y	Y	Y	Y	Y	Y	Y
Guo M	2020	Y	Y	Y	Y	N	N	Y	Y	Y
Wang	2022	Y	Y	Y	Y	Y	N	Y	Y	Y
Chen Y	2022	Y	Y	Y	Y	Y	N	Y	Y	Y
Sanowal	2023	Y	Y	Y	Y	N	N	Y	Y	Y
Wu	2023	Y	Y	Y	Y	Y	NA	Y	Y	Y
Lopes	2023	Y	Y	Y	Y	NA	NA	Y	Y	Y

**Q1**	Were the criteria for inclusion in the sample clearly defined?
**Q2**	Were the study subjects and the setting described in detail?
**Q3**	Was the exposure measured in a valid and reliable way?
**Q4**	Were objective, standard criteria used for measurement of the condition?
**Q5**	Were confounding factors identified?
**Q6**	Were strategies to deal with confounding factors stated?
**Q7**	Were the outcomes measured in a valid and reliable way?
**Q8**	Was appropriate statistical analysis used?

Y: Yes N: No NA: Not Available

S1 Fig. Meta-analysis of miscarriage according to the number of participants in the study: > 500 (yes) , ≤500 individuals (no)

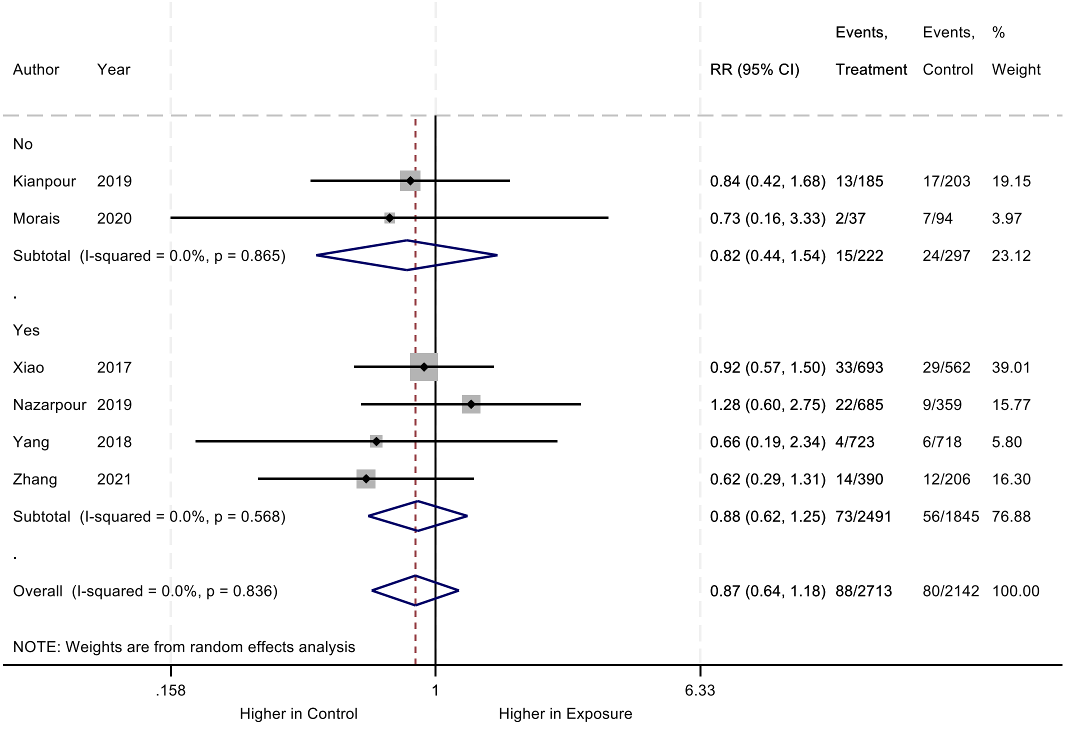

S2 Fig. Meta-analysis of miscarriage according to the studies that corrected UIC for

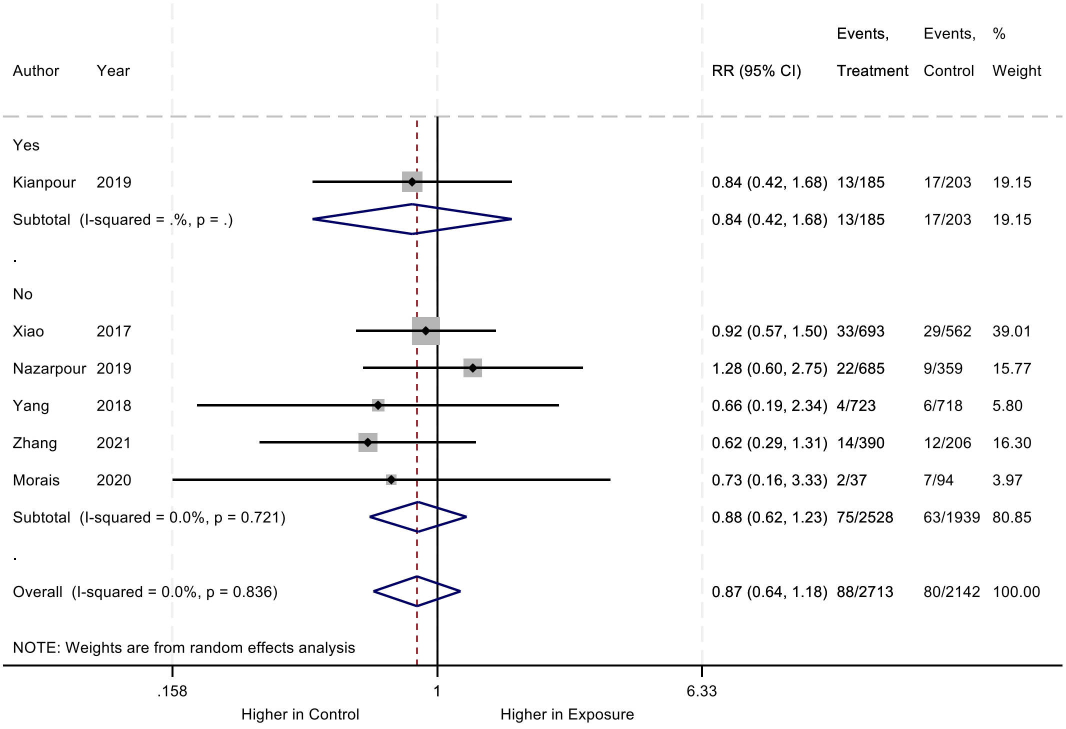

S3 Fig. Meta-analysis of maternal subclinical hypothyroidism according to the number of participants in the study: > 500 (yes) , ≤500 individuals (no)

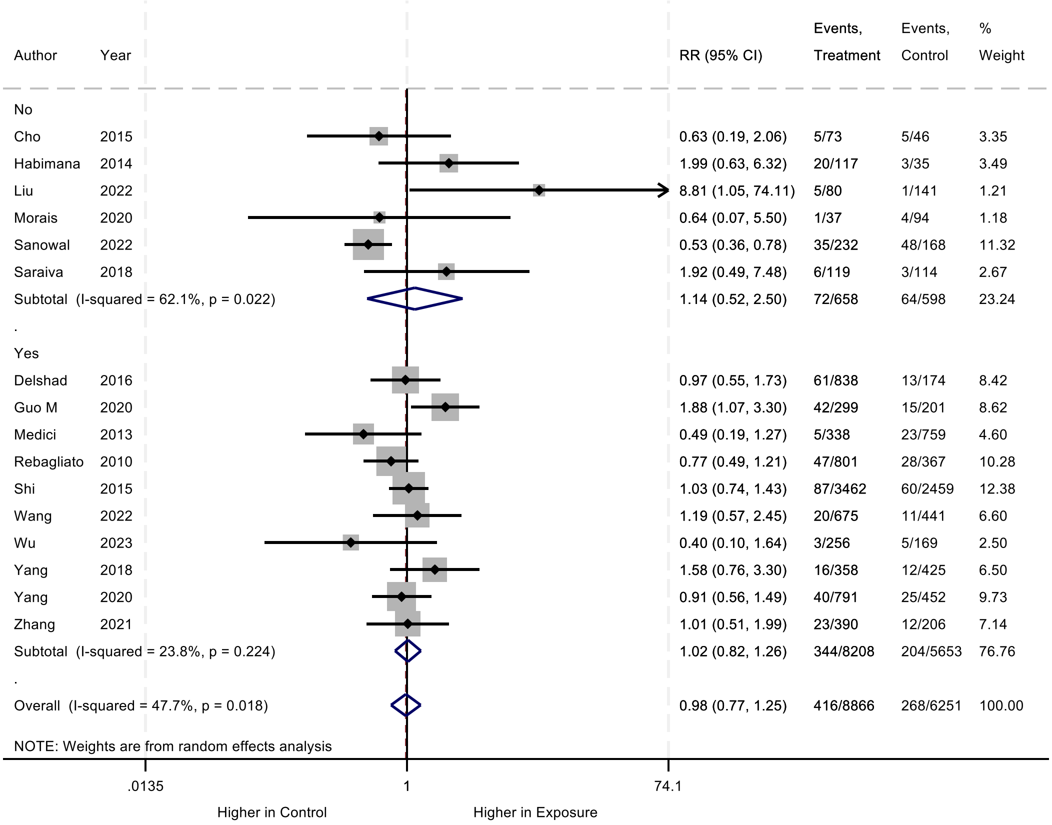
Author Year
RR (95% CI)
Events, Treatment
Events, Control
% Weight
No
Cho 2015 0.63 (0.19, 2.06) 5/73 5/46 3.35
Habimana 2014 1.99 (0.63, 6.32) 20/117 3/35 3.49
Liu 2022 8.81 (1.05, 74.11) 5/80 1/141 1.21
Morais 2020 0.64 (0.07, 5.50) 1/37 4/94 1.18
Sanowal 2022 0.53 (0.36, 0.78) 35/232 48/168 11.32
Saraiva 2018 1.92 (0.49, 7.48) 6/119 3/114 2.67
Subtotal (I-squared = 62.1%, p = 0.022) 1.14 (0.52, 2.50) 72/658 64/598 23.24
.
Yes
Delshad 2016 0.97 (0.55, 1.73) 61/838 13/174 8.42
Guo M 2020 1.88 (1.07, 3.30) 42/299 15/201 8.62
Medici 2013 0.49 (0.19, 1.27) 5/338 23/759 4.60
Rebagliato 2010 0.77 (0.49, 1.21) 47/801 28/367 10.28
Shi 2015 1.03 (0.74, 1.43) 87/3462 60/2459 12.38
Wang 2022 1.19 (0.57, 2.45) 20/675 11/441 6.60
Wu 2023 0.40 (0.10, 1.64) 3/256 5/169 2.50
Yang 2018 1.58 (0.76, 3.30) 16/358 12/425 6.50
Yang 2020 0.91 (0.56, 1.49) 40/791 25/452 9.73
Zhang 2021 1.01 (0.51, 1.99) 23/390 12/206 7.14
Subtotal (I-squared = 23.8%, p = 0.224) 1.02 (0.82, 1.26) 344/8208 204/5653 76.76
.
Overall (I-squared = 47.7%, p = 0.018) 0.98 (0.77, 1.25) 416/8866 268/6251 100.00
NOTE: Weights are from random effects analysis
.0135
1
74.1
Higher in Control
Higher in Exposure

S4 Fig. Meta-analysis of maternal subclinical hypothyroidism according to the studies that corrected UIC for urinary creatinine

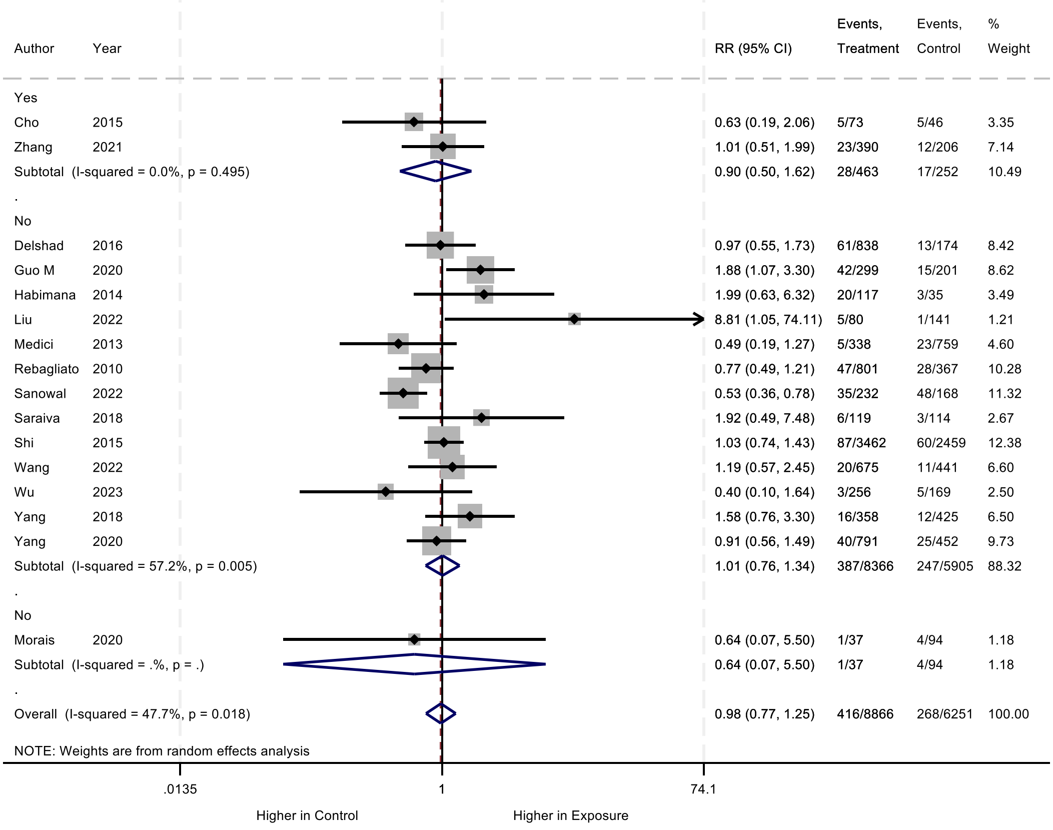

S5 Fig. Meta-analysis of thyroid volume

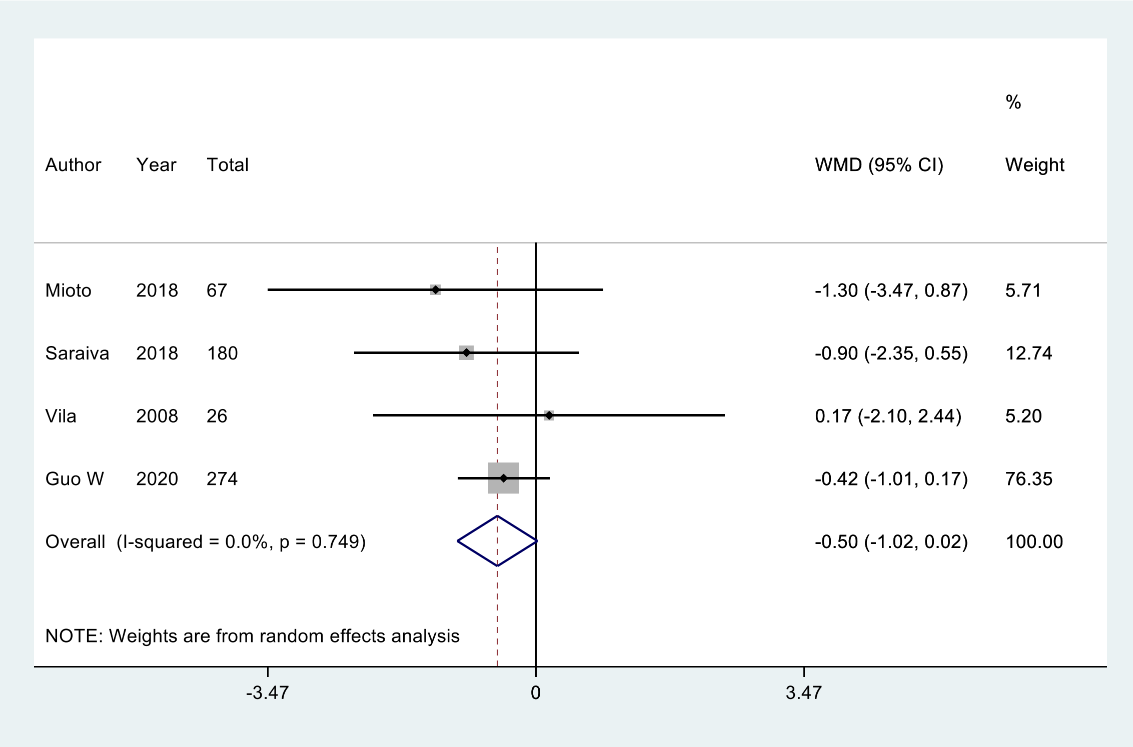

S6 Fig. Meta-analysis of maternal TSH measurement by comparing the exposure effect according to published (no) vs. imputed data (yes)

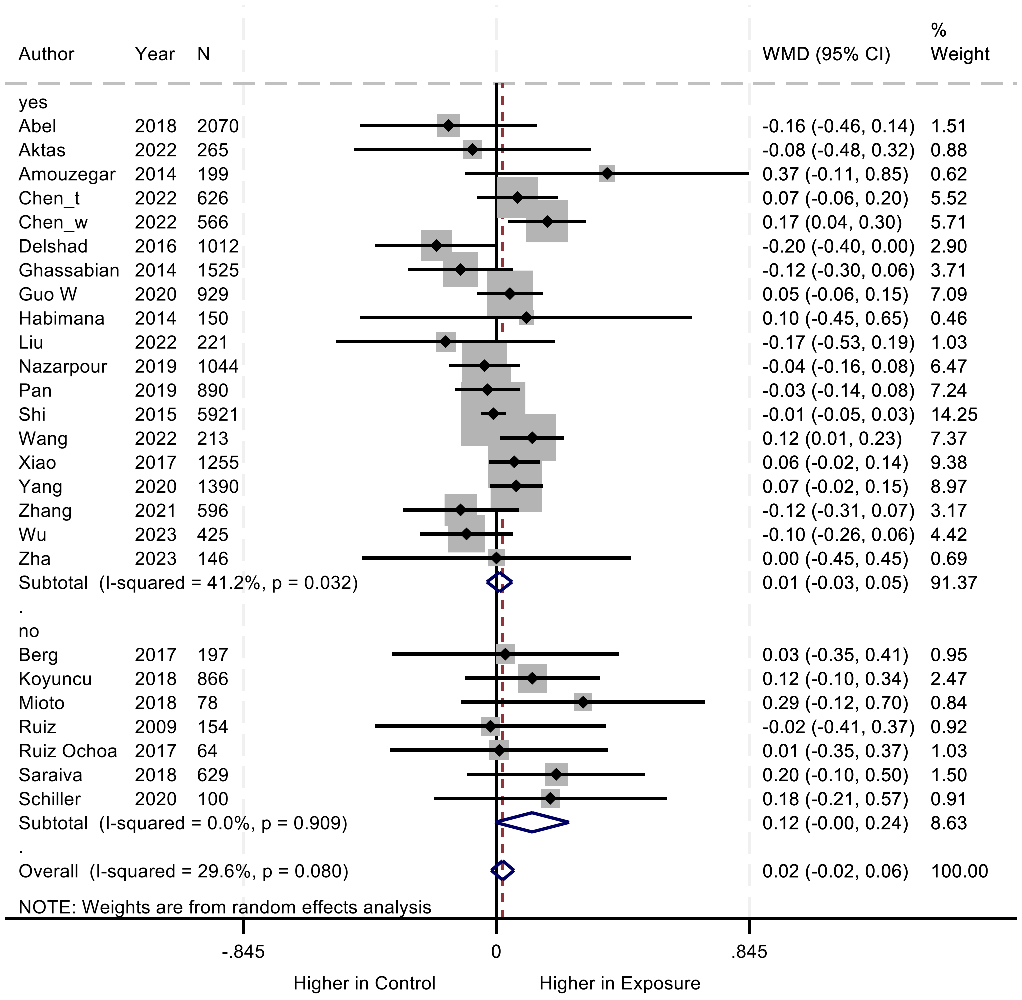

S7 Fig. Meta-analysis of maternal TSH measurement by comparing the exposure effect according to the number of participants in the study: > 500 (yes) , ≤500 individuals (no)

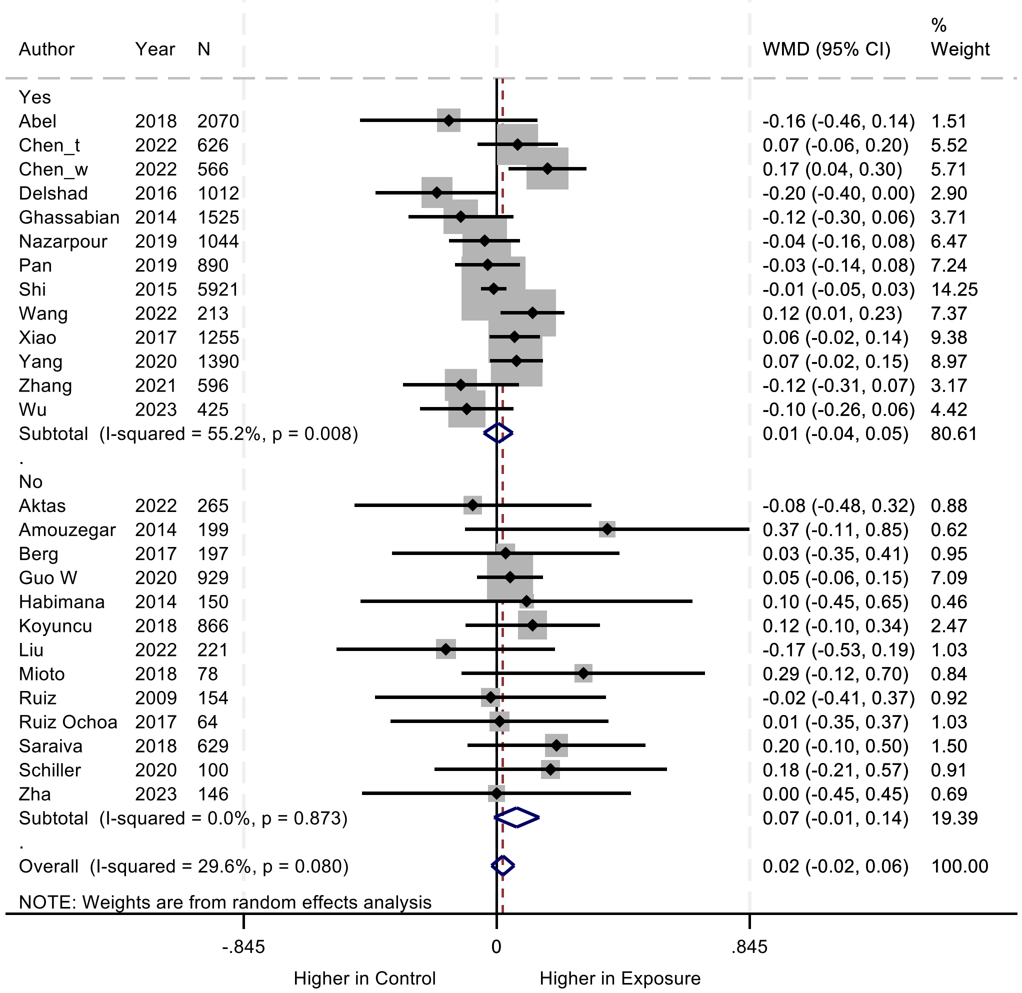

S8 Fig. Meta-analysis of maternal TSH measurement by comparing the exposure effect according to the studies that corrected UIC for urinary creatinine: yes vs no

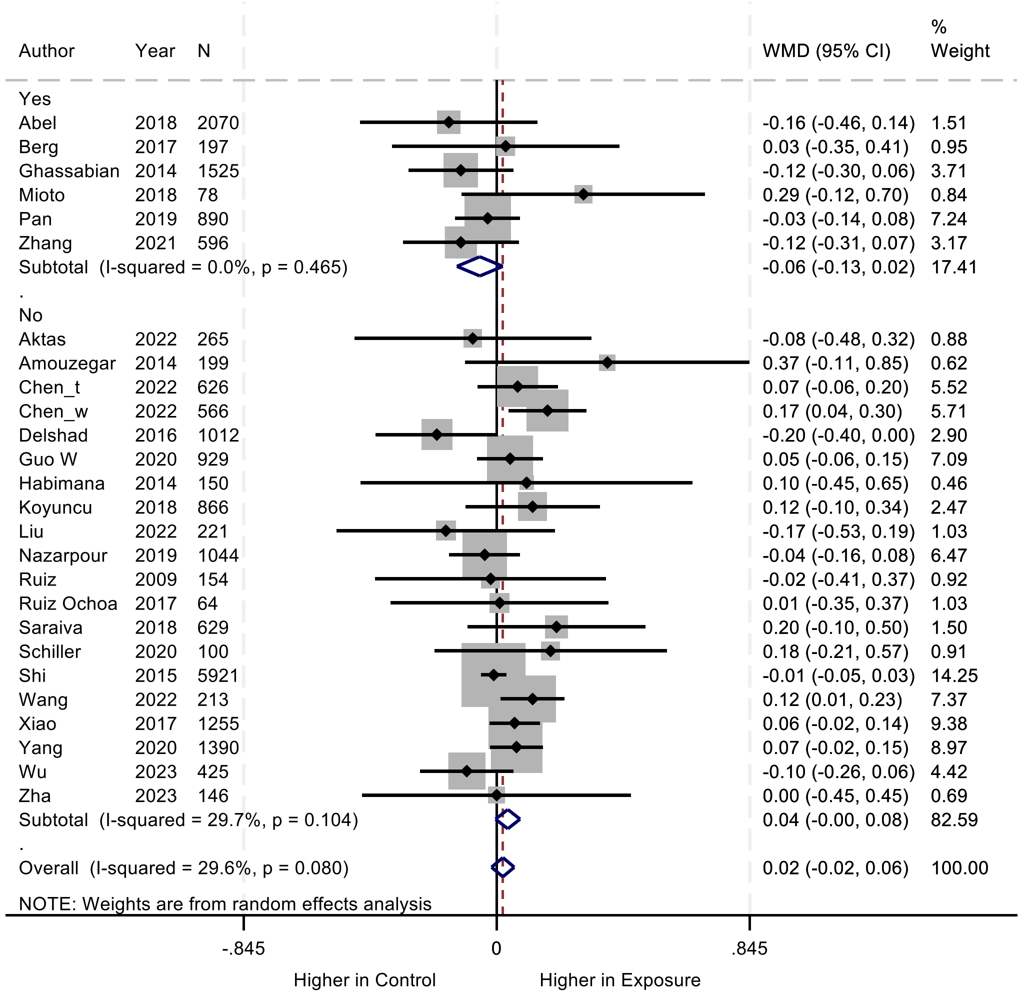

S9 Fig. Meta-analysis of maternal TSH measurement by comparing the exposure effect according to the gestational trimester

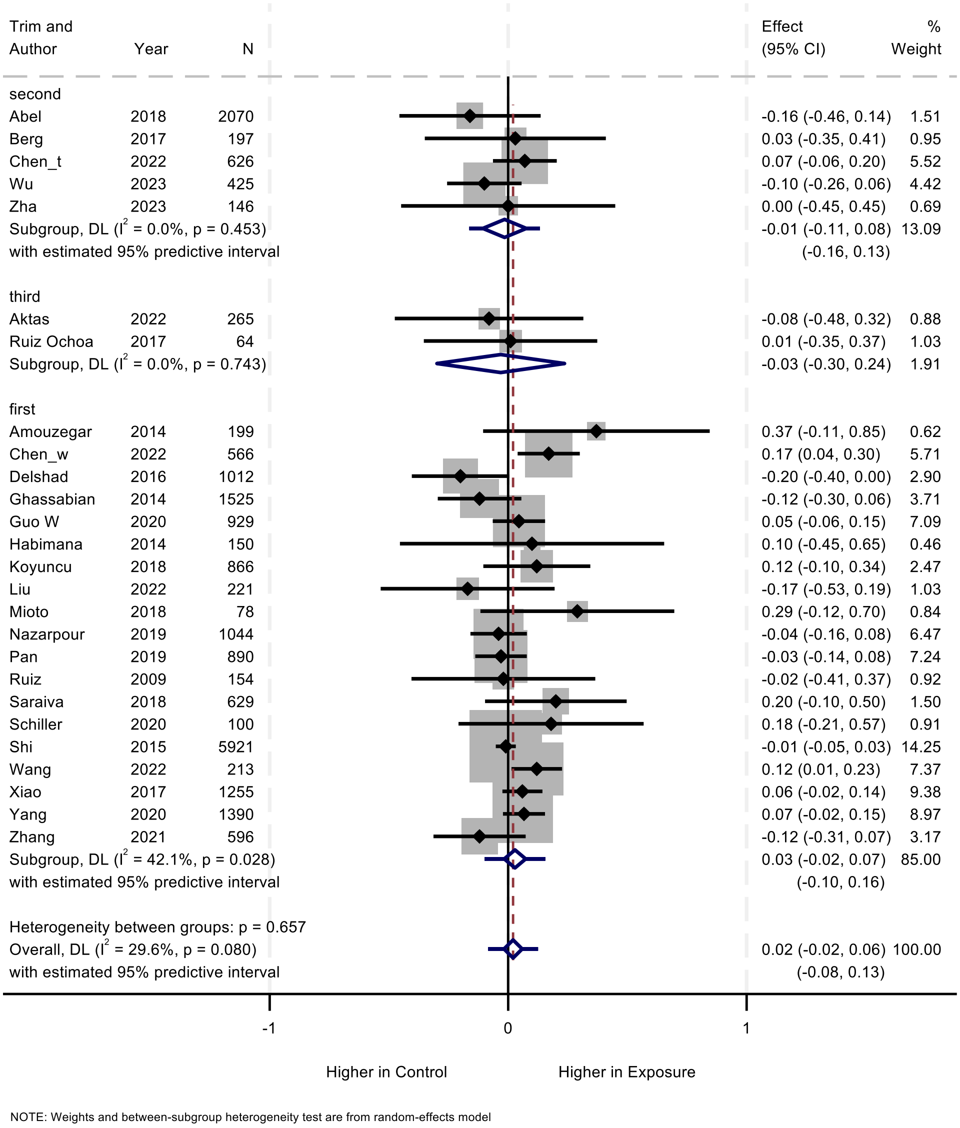

S10 Fig. Meta-analysis of preterm birth, by comparing the exposure effect according to the number of participants in the study: > 500 (yes) , ≤500 individuals (no)

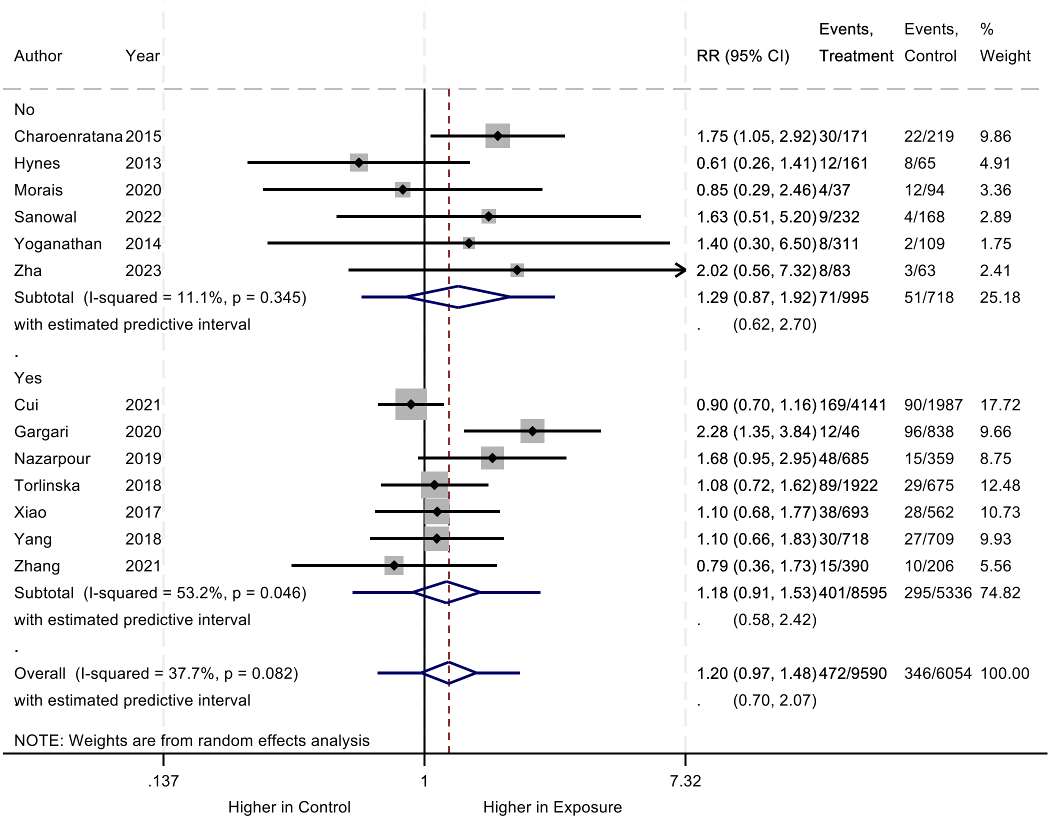

S11 Fig. Meta-analysis of preterm birth by comparing the exposure effect according to the studies that corrected UIC for urinary creatinine: yes vs no

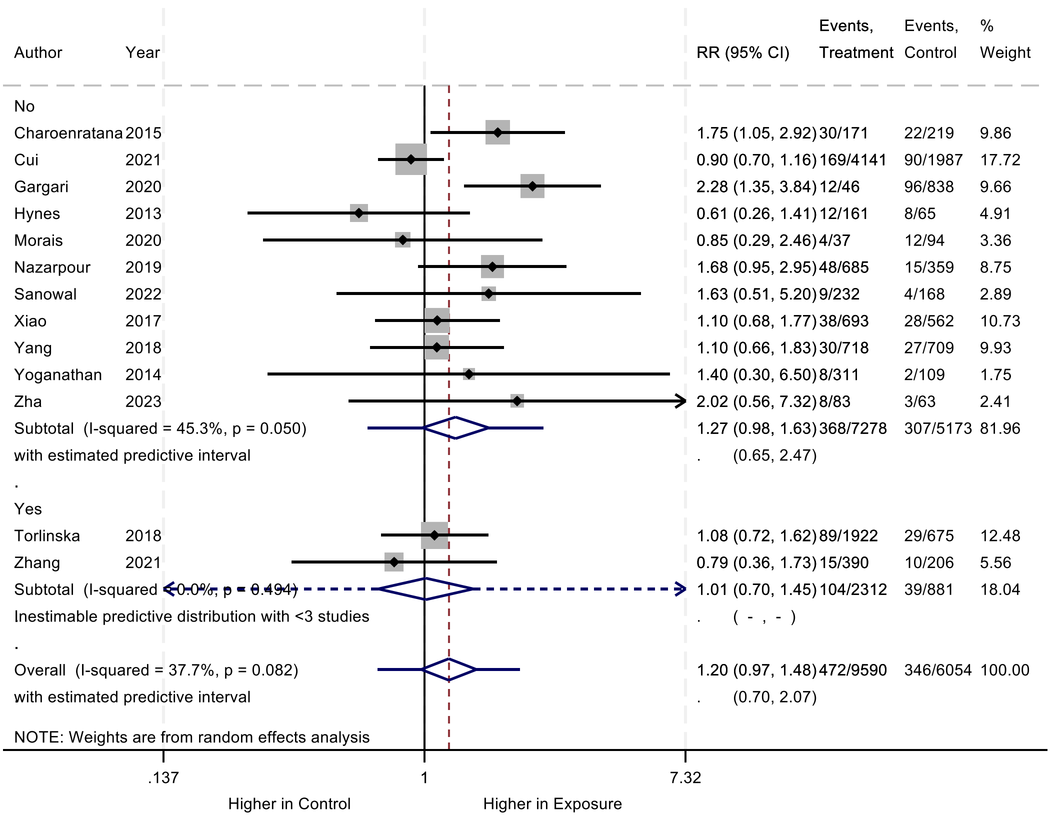

S12 Fig. Meta-analysis of low birthweight by comparing the exposure effect according to the number of participants in the study: ≥ 500 (yes) ... <500 individuals (no)

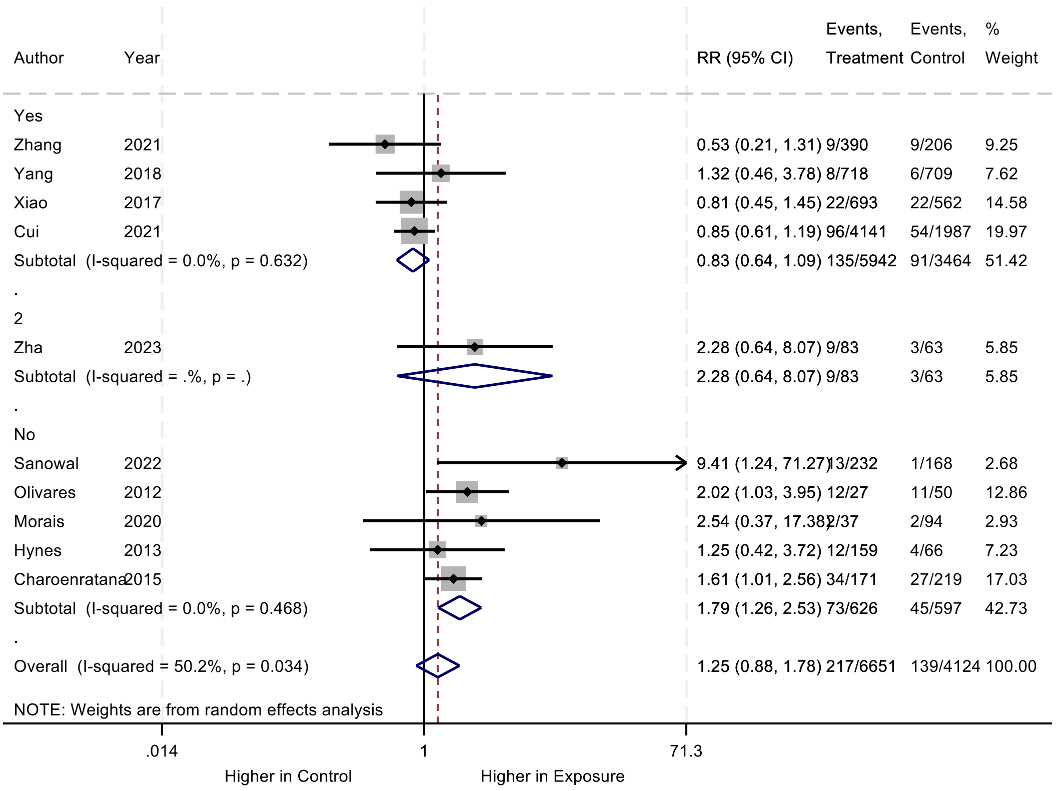

S13 Fig. Meta-analysis of low birthweight by comparing the exposure effect according to the studies that corrected UIC for urinary creatinine: yes vs no

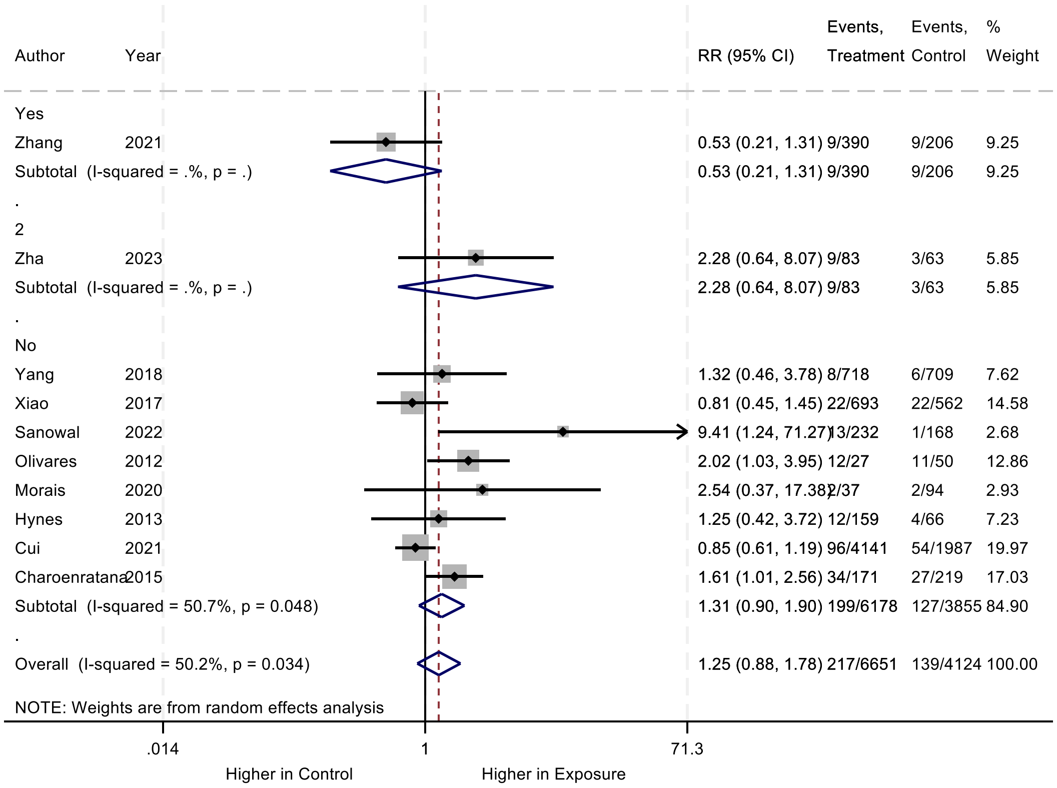

S14 Fig. Meta-analysis of elevated neonatal TSH

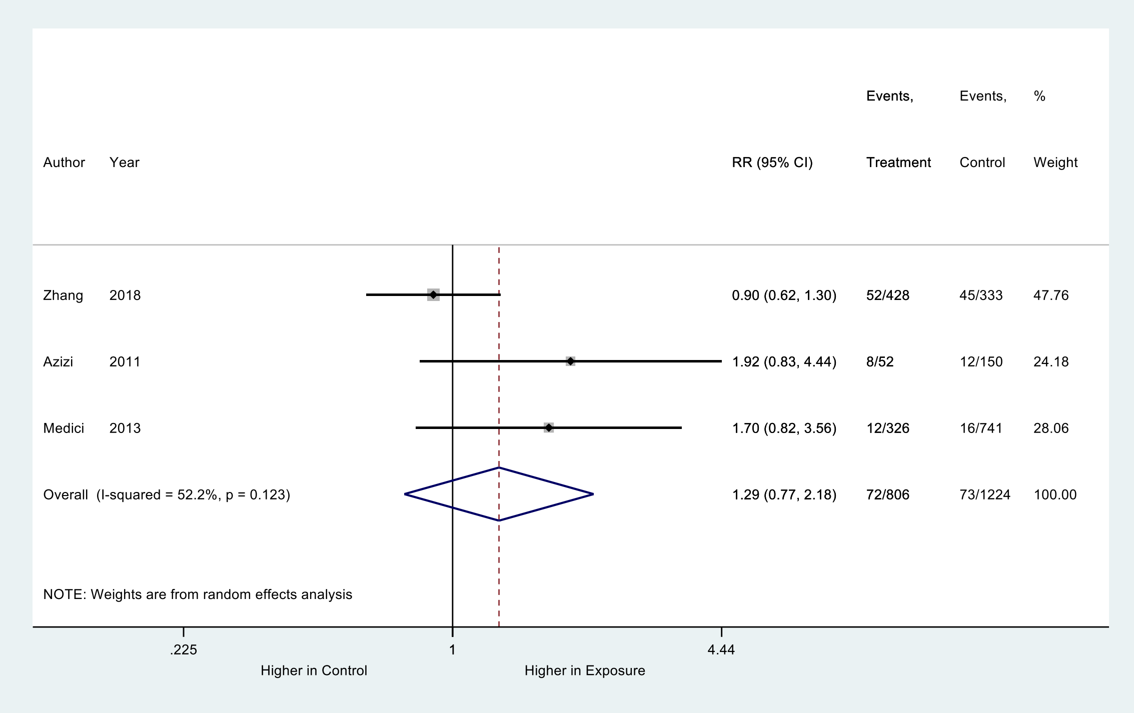

S15 Fig: Funnel plot of Preterm birth

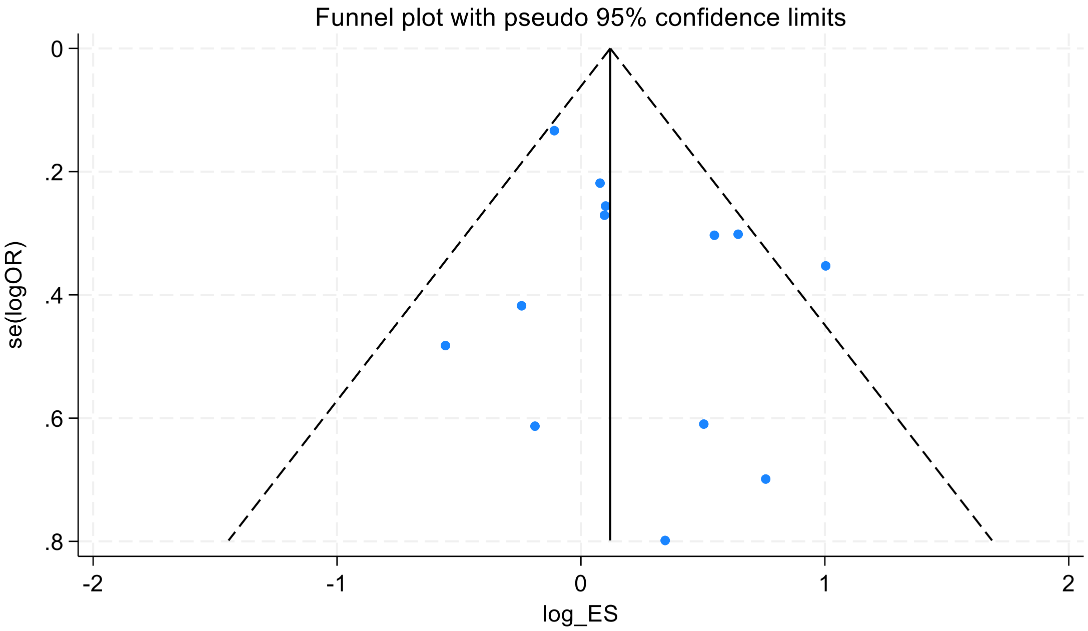

S16 Fig. Egger test of preterm birth

```
.
Number of studies =  13                                 Root MSE      =   1.213
------------------------------------------------------------------------------
     Std_Eff | Coefficient  Std. err.      t    P>|t|     [95% conf. interval]
-------------+----------------------------------------------------------------
       slope |  -.1098122   .2061139    -0.53   0.605    -.5634658    .3438414
        bias |   .9017744   .7114728     1.27   0.231    -.6641667    2.467715
------------------------------------------------------------------------------

Test of H0: no small-study effects          P = 0.231
```

S17 Fig. Meta-regression analysis of frequency prematurity for the covariates study design, iodine status and UIC used as control group.

```
. metareg logrr  studydesign  iodinestatus  iodo, wsse( selogrr)

Meta-regression                                       Number of obs  =        11
REML estimate of between-study variance               tau2           =     .0525
% residual variation due to heterogeneity             I-squared_res  =    43.12%
Proportion of between-study variance explained        Adj R-squared  =    -1.23%
Joint test for all covariates                         Model F(3,7)   =      0.50
With Knapp-Hartung modification                       Prob > F       =    0.6964
------------------------------------------------------------------------------
       logrr | Coefficient  Std. err.      t    P>|t|     [95% conf. interval]
-------------+----------------------------------------------------------------
 studydesign |   .5598443   .7206115     0.78   0.463    -1.144131     2.26382
iodinestatus |  -.1888096   .2961974    -0.64   0.544    -.8892052    .5115859
        iodo |  -.3195257   .3318017    -0.96   0.368    -1.104112    .4650606
       _cons |   .3851391   .9199786     0.42   0.688    -1.790265    2.560543
------------------------------------------------------------------------------
```

S18 Fig. Meta-regression analysis of frequency prematurity for the covariate age

```
.  metareg logrr  age, wsse( selogrr)

Meta-regression                                       Number of obs  =        12
REML estimate of between-study variance               tau2           =    .06778
% residual variation due to heterogeneity             I-squared_res  =    44.15%
Proportion of between-study variance explained        Adj R-squared  =   -15.84%
With Knapp-Hartung modification
------------------------------------------------------------------------------
       logrr | Coefficient  Std. err.      t    P>|t|     [95% conf. interval]
-------------+----------------------------------------------------------------
         age |  -.0394804    .104699    -0.38   0.714    -.2727642    .1938034
       _cons |   1.295504   2.966178     0.44   0.672    -5.313552    7.904559
------------------------------------------------------------------------------
```

S19 Fig. Meta-regression analysis of frequency of low birthweight for the covariates study design, iodine status and UIC used as control group

```
. metareg logrr  studydesign  iodinestatus  iodo, wsse( selogrr)

Meta-regression                                      Number of obs  =       9
REML estimate of between-study variance              tau2           =       0
% residual variation due to heterogeneity            I-squared_res  =   0.00%
Proportion of between-study variance explained       Adj R-squared  = 100.00%
Joint test for all covariates                        Model F(3,5)   =    3.87
With Knapp-Hartung modification                      Prob > F       =  0.0898
```

logrr	Coefficient	Std. err.	t	P>|t|	[95% conf.	interval]
studydesign	1.41927	1.21786	1.17	0.296	-1.71134	4.549879
iodinestatus	.9849188	.6590672	1.49	0.195	-.7092675	2.679105
iodo	.2518653	.7083182	0.36	0.737	-1.568925	2.072655
_cons	-3.069931	1.773388	-1.73	0.144	-7.62857	1.488707

S20 Fig. Meta-regression analysis of frequency of low birthweight for the covariate age

```
. metareg logrr  age, wsse( selogrr)

Meta-regression                                      Number of obs  =       9
REML estimate of between-study variance              tau2           =  .02531
% residual variation due to heterogeneity            I-squared_res  =  13.76%
Proportion of between-study variance explained       Adj R-squared  =  73.11%
With Knapp-Hartung modification
```

logrr	Coefficient	Std. err.	t	P>|t|	[95% conf.	interval]
age	-.1793767	.0899305	-1.99	0.086	-.3920285	.0332751
_cons	5.167323	2.530377	2.04	0.080	-.8160676	11.15071

S21 Fig. Meta-regression analysis of frequency of maternal hypothyroidism for the covariates study design, iodine status and UIC used as control group

```
. metareg logrr  studydesign  iodinestatus  iodo, wsse( selogrr)

Meta-regression                                       Number of obs  =       7
REML estimate of between-study variance               tau2           =       0
% residual variation due to heterogeneity             I-squared_res  =   0.00%
Proportion of between-study variance explained        Adj R-squared  = 100.00%
Joint test for all covariates                         Model F(3,3)   =    1.88
With Knapp-Hartung modification                       Prob > F       =  0.3084
```

logrr	Coefficient	Std. err.	t	P>|t|	[95% conf.	interval]
studydesign	-.1776611	.7996993	-0.22	0.838	-2.722661	2.367339
iodinestatus	-.8903353	.42412	-2.10	0.127	-2.240074	.4594039
iodo	-.1014427	.4307994	-0.24	0.829	-1.472439	1.269553
_cons	1.73012	1.781473	0.97	0.403	-3.939323	7.399563

S22 Fig. Meta-regression analysis of frequency of maternal hypothyroidism for the covariate age

```
. metareg logrr  age, wsse( selogrr)

Meta-regression                                       Number of obs  =       5
REML estimate of between-study variance               tau2           = .009571
% residual variation due to heterogeneity             I-squared_res  =   7.67%
Proportion of between-study variance explained        Adj R-squared  =  89.67%
With Knapp-Hartung modification
```

logrr	Coefficient	Std. err.	t	P>|t|	[95% conf.	interval]
age	.4733178	.3258396	1.45	0.242	-.5636492	1.510285
_cons	-12.90718	8.863617	-1.46	0.241	-41.11516	15.3008

S23 Fig. Meta-regression analysis of frequency of maternal subclinical hypothyroidism for the covariates study design, iodine status and UIC used as control group

```
. metareg logrr  studydesign  iodinestatus  iodo, wsse( selogrr)

Meta-regression                                       Number of obs  =       15
REML estimate of between-study variance               tau2           =   .01214
% residual variation due to heterogeneity             I-squared_res  =   25.27%
Proportion of between-study variance explained        Adj R-squared  =   84.54%
Joint test for all covariates                         Model F(3,11)  =     1.94
With Knapp-Hartung modification                       Prob > F       =   0.1816

------------------------------------------------------------------------------
       logrr | Coefficient  Std. err.      t    P>|t|     [95% conf. interval]
-------------+----------------------------------------------------------------
 studydesign |  -.0197039   .2261783    -0.09   0.932    -.5175189    .4781112
iodinestatus |   -.458892   .2230574    -2.06   0.064     -.949838     .032054
        iodo |   -.219903   .3192086    -0.69   0.505    -.9224764    .4826703
       _cons |   .9771093   .7386951     1.32   0.213    -.6487477    2.602966
------------------------------------------------------------------------------
```

S24 Fig. Meta-regression analysis of frequency of maternal subclinical hypothyroidism for the covariate age

```
. metareg logrr  age, wsse( selogrr)
numerical derivatives are approximate
nearby values are missing

Meta-regression                                       Number of obs  =       12
REML estimate of between-study variance               tau2           =        0
% residual variation due to heterogeneity             I-squared_res  =    1.63%
Proportion of between-study variance explained        Adj R-squared  =       .%
With Knapp-Hartung modification

------------------------------------------------------------------------------
       logrr | Coefficient  Std. err.      t    P>|t|     [95% conf. interval]
-------------+----------------------------------------------------------------
         age |  -.1125723    .0596906   -1.89   0.089    -.2455713    .0204267
       _cons |    3.22903    1.730772    1.87   0.092    -.6273714    7.085432
------------------------------------------------------------------------------
```

S25 Fig. Meta-regression analysis of frequency of maternal TSH for the covariates study design, iodine status and UIC used as control group

```
.
. . xi:metareg   _ES studydesign  iodinestatus  iodo, wsse( _seES)

Meta-regression                                       Number of obs  =       25
REML estimate of between-study variance               tau2           =  .002888
% residual variation due to heterogeneity             I-squared_res  =   34.70%
Proportion of between-study variance explained        Adj R-squared  =  -10.49%
Joint test for all covariates                         Model F(3,21)  =     0.90
With Knapp-Hartung modification                       Prob > F       =   0.4586

------------------------------------------------------------------------------
         _ES | Coefficient  Std. err.      t    P>|t|     [95% conf. interval]
-------------+----------------------------------------------------------------
 studydesign |   .0519506    .0493753    1.05   0.305     -.050731    .1546322
iodinestatus |  -.0770458    .0743947   -1.04   0.312     -.231758    .0776665
        iodo |   .0129047    .0782207    0.16   0.871    -.1497642    .1755736
       _cons |  -.0062123    .1881531   -0.03   0.974     -.397498    .3850734
------------------------------------------------------------------------------
```

S26 Fig. Meta-regression analysis of frequency of maternal TSH for the covariate age

```
. metareg  _ES age, wsse( _seES)

Meta-regression                                    Number of obs  =       23
REML estimate of between-study variance            tau2           = .005345
% residual variation due to heterogeneity          I-squared_res  =  32.55%
Proportion of between-study variance explained     Adj R-squared  =  -1.22%
With Knapp-Hartung modification
```

_ES	Coefficient	Std. err.	t	P>|t|	[95% conf. interval]	
age	-.0199896	.0199939	-1.00	0.329	-.0615692	.02159
_cons	.5820817	.5670992	1.03	0.316	-.5972655	1.761429

SEARCH STRATEGIES

Medline and CENTRAL-Cochrane Strategies:

Search on the Medline (PubMed) and CENTRAL-Cochrane platforms:

#1 “Iodine"[Mesh] OR (Iodine-127) OR (Iodine 127) OR (Urinary Iodine) OR (Iodine deficient) OR (Iodine deficiency) OR (Iodine sufficient) OR (Iodine levels) OR (Levels of iodine) OR (Iodine sources) OR (Iodine status) OR (Iodine stores) OR (Iodine concentration) OR (Iodine intake) OR (Intake of iodine) OR (Urine iodine concentration) OR (Urinary iodine concentration) OR (Renal iodide clearance) OR (Median urinary iodine concentration) OR (Urinary iodine excretion) OR (24-hour urinary iodine excretion) OR (Urinary iodine) OR (Urinary iodine analysis) OR (urinary iodine testing) OR (Dosage of iodine) OR (Loss of iodine) OR (Urinary iodine values) OR (Iodine-containing supplements) OR (Iodine supplementation) OR (Iodine supplement) OR (Supplementary iodine intake) OR (Iodine nutrition) OR (Status of iodine nutrition) OR (Iodine nutritional status) OR (Iodine metabolism) OR (Thyroid iodide clearance) OR (Absolute iodide uptake) OR (Oral load of iodine) OR (Iodine-rich food) OR (Dietary iodine intake) OR (Dietary iodine sources) OR (Dietary iodine requirement) OR (Salt iodization) OR (Iodized salt) OR (Iodine/day) OR (Level of Iodine Intake) OR (Iodine deficient area) OR (Iodine-sufficient area) OR (Iodine-sufficient populations) OR (Fetal iodine requirements)

#2 "Pregnancy"[Mesh] OR (Pregnancies) OR (Gestation) OR (Maternal Iodine) OR (Pregnant women) OR (Pregnant) OR (Lactating women) OR (Maternal iodine status) OR (Maternal iodine intake) OR (Postpartum) OR (Pregnant patient)

Embase Strategy

Search on the Embase (Elsevier) platform:

#1 'iodine'/exp OR '127 I' OR '127 I iodine' OR '127 iodine' OR '127I' OR 'hormonal iodine' OR 'I 127' OR 'inorganic iodine' OR 'iod sol' OR 'iodine 127' OR 'iodine I 127' OR 'iodine isotope' OR 'iodine isotopes' OR 'iodine mixture' OR 'iodine solution' OR 'iodine tincture' OR 'iodium' OR 'iosal' OR 'J 127' OR 'jodium' OR 'medadine' OR 'neo hydriol fluid' OR 'neo hydriol viscous' OR 'sublimed iodine' OR 'tincture of iodine' OR ‘iodine-127’ OR ‘iodine 127’ OR ‘urinary iodine’ OR ‘iodine deficient’ OR ‘iodine deficiency’ OR ‘iodine sufficient’ OR ‘iodine levels’ OR ‘levels of iodine’ OR ‘iodine sources’ OR ‘iodine status’ OR ‘iodine stores’ OR ‘iodine concentration’ OR ‘iodine intake’ OR ‘intake of iodine’ OR ‘urine iodine concentration’ OR ‘urinary iodine concentration’ OR ‘renal iodide clearance’ OR ‘median urinary iodine concentration’ OR ‘urinary iodine excretion’ OR ‘24-hour urinary iodine excretion’ OR ‘urinary iodine’ OR ‘urinary iodine analysis’ OR ‘urinary iodine testing’ OR ‘dosage of iodine’ OR ‘loss of iodine’ OR ‘urinary iodine values’ OR ‘iodine-containing supplements’ OR ‘iodine supplementation’ OR ‘iodine supplement’ OR ‘supplementary iodine intake’ OR ‘iodine nutrition’ OR ‘status of iodine nutrition’ OR ‘iodine nutritional status’ OR ‘iodine metabolism’ OR ‘thyroid iodide clearance’ OR ‘absolute iodide uptake’ OR ‘oral load of iodine’ OR ‘iodine-rich food’ OR ‘dietary iodine intake’ OR ‘dietary iodine sources’ OR ‘dietary iodine requirement’ OR ‘salt iodization’ OR ‘iodized salt’ OR ‘iodine/day’ OR ‘level of Iodine Intake’ OR ‘iodine deficient area’ OR ‘iodine-sufficient area’ OR ‘iodine-sufficient populations’ OR ‘fetal iodine requirements’

#2 'pregnancy'/exp OR 'child bearing' OR 'childbearing' OR 'gestation' OR 'gravidity' OR 'intrauterine pregnancy' OR 'labor presentation' OR 'labour presentation' OR 'pregnancy maintenance' OR 'pregnancy trimesters' OR ‘pregnancies’ OR ‘maternal iodine’ OR ‘pregnant women’ OR ‘pregnant’ OR ‘lactating women’ OR ‘maternal iodine status’ OR ‘maternal iodine intake’ OR ‘postpartum’ OR ‘pregnant patient’

LILACS Strategy

Search on the LILACS (BVS) platform:

#1 MH:"Iodo" OR (Iodo-127) OR MH:D01.268.380.400$ OR MH:SP4.011.097.063.999$ OR MH:SP4.021.202.133.794.825$

2 MH:"Gravidez" OR (Gestação) OR MH:G08.686.784.769$

References of the excluded studies

1) Ardawi MSM, Nasrat HA, Mustafa BE. Urinary iodine excretion and maternal thyroid function. During pregnancy and postpartum. Saudi Med J 2002; 23(4):413-22.
2) Azizi F, Aminorroya A, Hedayati M, et al. Urinary iodine excretion in pregnant women residing in areas with adequate iodine intake. Public Health Nutr 2003;6(1):95-8; doi: 10.1079/PHN2002366.
3) Blumenthal N, Byth K, Eastman CJ. Iodine Intake and Thyroid Function in Pregnant Women in a Private Clinical Practice in Northwestern Sydney before Mandatory Fortification of Bread with Iodised Salt. Thyroid Res 2012;2012:798963; doi: 10.1155/2012/798963.
4) Brucker-Davis F, Ferrari P, Gal J, et al. Iodine status has no impact on thyroid function in early healthy pregnancy. J Thyroid Res. 2012;2012:168764; doi: 10.1155/2012/168764.
5) Castillo C, Lustig N, Margozzini P, et al. Thyroid-Stimulating Hormone Reference Ranges in the First Trimester of Pregnancy in an Iodine-Sufficient Country. Endocrinol Metab (Seoul) 2018;33(4):466-472; doi: 10.3803/EnM. 2018.33.4.466.
6) Chakraborty I, Chatterjee S, Bhadra D, et al. Iodine deficiency disorders among the pregnant women in a rural hospital of West Bengal. Indian J Med Res 2006;123(6):825-829.
7) Chakraborty I, Mazumdar P, Chakraborty PS, et al. Iodine deficiency disorder among pregnant women in a tertiary care hospital of Kolkata, India. Southeast Asian J Trop Med Public Health 2010;41(4):989-995.
8) Chaudhary LN, Khatiwada S, Geal B, et al. Iodine and Thyroid Function Status, and Anti-thyroid Peroxidase Antibody among Pregnant Women in Eastern Nepal. J Nepal Health Res Counc 2017;15(2):114-119; doi: 10.3126/jnhrc. v15i2.18182.

9) Chen W, Sang Z, Tan L, et al. Neonatal thyroid function born to mothers living with long-term excessive iodine intake from drinking water. Clin Endocrinol (Oxf) 2015;83(3):399-404; doi: 10.1111/cen.12625.
10) Chinyanga EA, Chidede O, Machisvo A, et al. Urinary iodine excretion in pregnant women as an index of the impact of a national iodization programme. Cent Afr J Med. 2006;52(7-8):78-83; doi: 10.4314/cajm.v52i7-8.62585.
11) Chinyanga EA, Dako DY. Profile of thyroid function and urinary iodine excretion of pregnant women attending Harare Central Hospital antenatal clinic. Cent Afr J Med 1989;35(5):396-400.
12) Costeira MJ, Oliveira P, Ares S, et al. Iodine status of pregnant women and their progeny in the Minho Region of Portugal. Thyroid 2009;19(2):157-163; doi: 10.1089/thy.2008.0249.
13) Cromie KJ, Threapleton DE, Snart CJP, et al. Maternal iodine status in a multi-ethnic UK birth cohort: associations with autism spectrum disorder. BMC Pediatr 2020;20(1):544; doi: 10.1186/s12887-020-02440-y.
14) Cuellar-Rufino S, Navarro-Meza M, García-Solís P, et al. Iodine levels are associated with oxidative stress and antioxidant status in pregnant women with hypertensive disease. Nutr Hosp 2017;34(3):661-666; doi: 10.20960/ nh.460.
15) Das SC, Isichei UP, Mohammed AZ, et al. Impact of iodine deficiency on thyroid function in pregnant African women - A possible factor in the genesis of 'small for dates' babies. Indian J Clin Biochem 2005;20(2):35-42; doi: 10.1007/ BF02867398.
16) De Zoysa E, Hettiarachchi M, Liyanage C. Urinary iodine and thyroid determinants in pregnancy: a follow up study in Sri Lanka. BMC Pregnancy Childbirth 2016;16(1):303; doi: 10.1186/s12884-016-1093-7.
17) Dodd NS, Madan J. Iodine status in pregnancy. Asia Pac J Clin Nutr 1993; 2(3):119-123
18) Dold S, Zimmermann MB, Jukic T, et al. Universal Salt Iodization Provides Sufficient Dietary Iodine to Achieve Adequate Iodine Nutrition during the First 1000 Days: A Cross-Sectional Multicenter Study. J Nutr 2018;148(4): 587-598; doi: 10.1093/jn/nxy015.
19) Domínguez I, Reviriego S, Rojo-Martínez G, et al. Iodine deficiency and thyroid function in healthy pregnant women. Med Clin (Barc) 2004;122(12):449-453; doi: 10.1016/s0025-7753(04)74269-4.
20) Elahi S, Rizvi NB, Nagra SA. Iodine deficiency in pregnant women of Lahore. J Pak Med Assoc. 2009;59(11):741-743.
21) Elnagar B, Eltom A, Wide L, et al. Iodine status, thyroid function and pregnancy: study of Swedish and Sudanese women. J Clin Nutr 1998;52(5): 351-355; doi: 10.1038/sj.ejcn.1600563.
22) Eltom A, Elnagar B, Elbagir M, et al. Thyroglobulin in serum as an indicator of iodine status during pregnancy. Scand J Clin Lab Invest 2000;60(1):1-7; doi: 10.1080/00365510050184985.

23) Eltom A, Elnagar B, Gebre-Medhin M. Thyroid hormones and iodine status in Sudanese pregnant women with goitre. Int J Food Sci Nutr 1999;50(2):105-109; doi: 10.1080/096374899101292.
24) Farebrother J, Zimmermann MB, Abdallah F, et al. Effect of Excess Iodine Intake from Iodized Salt and/or Groundwater Iodine on Thyroid Function in Nonpregnant and Pregnant Women, Infants, and Children: A Multicenter Study in East Africa. Thyroid 2018;28(9):1198-1210; doi: 10.1089/thy. 2018.0234.
25) Fereja M, Gebremedhin S, Gebreegziabher T, et al. Prevalence of iodine deficiency and associated factors among pregnant women in Ada district, Oromia region, Ethiopia: a cross- sectional study. BMC Pregnancy Childbirth 2018;18(1):257; doi: 10.1186/s12884-018-1905-z.
26) Filteau SM, Sullivan KR, Anwar US, et al. Iodine deficiency alone cannot account for goitre prevalence among pregnant women in Modhupur, Bangladesh. Eur J Clin Nutr 1994;48(4):293-302.
27) Fister P, Gaberscek S, Zaletel K, et al. Thyroid volume changes during pregnancy and after delivery in an iodine-sufficient Republic of Slovenia. Eur J Obstet Gynecol Reprod Biol. 2009;145(1):45-48; doi: 10.1016/j.ejogrb. 2009.03.022.
28) Fuse Y, Ohashi T, Yamaguchi S, et al. Iodine status of pregnant and postpartum Japanese women: effect of iodine intake on maternal and neonatal thyroid function in an iodine-sufficient area. J Clin Endocrinol Metab 2011;96(12):3846-3854; doi: 10.1210/jc.2011-2180.
29) Fuse Y, Shishiba Y, Irie M. Gestational changes of thyroid function and urinary iodine in thyroid antibody-negative Japanese women. Endocr J 2013;60(9): 1095-1096; doi: 10.1507/endocrj.ej13-0184.
30) Gaitan E, Cooksey RC, Meydrech EF, et al. Thyroid function in neonates from goitrous and nongoitrous iodine-sufficient areas. J Clin Endocrinol Metab 1989;69(2):359-363; doi: 10.1210/jcem-69-2-359.
31) Gietka-Czernel M, Dębska M, Kretowicz P, et al. Iodine status of pregnant women from central Poland ten years after introduction of iodine prophylaxis programme. Endokrynol Pol 2010;61(6):646-651.
32) Gowachirapant S, Melse-Boonstra A, Winichagoon P, et al. Overweight increases risk of first trimester hypothyroxinaemia in iodine-deficient pregnant women. Matern Child Nutr 2014;10(1):61-71; doi: 10.1111/mcn. 12040.
33) Grau G, Aguayo A, Vela A, et al. Normal intellectual development in children born from women with hypothyroxinemia during their pregnancy. J Trace Elem Med Biol 2015;31:18-24; doi: 10.1016/j.jtemb.2015.02.004.
34) Guan H, Li C, Li Y, et al. High iodine intake is a risk factor of post-partum thyroiditis: result of a survey from Shenyang, China. Endocrinol Invest 2005;28(10):876-881; doi: 10.1007/BF03345318.
35) Gunnarsdottir I, Gustavsdottir AG, Steingrimsdottir L, et al. Iodine status of pregnant women in a population changing from high to lower fish and milk

consumption. Public Health Nutr 2013;16(2):325-329; doi: 10.1017/ S1368980012001358.
36) Habimana L, Twite KE, Wallemacq P, et al. Iodine and iron status of pregnant women in Lubumbashi, Democratic Republic of Congo. Public Health Nutr 2013;16(8):1362-1370; doi: 10.1017/S1368980012005484.
37) Henjum S, Aakre I, Lilleengen AM, et al. Suboptimal Iodine Status among Pregnant Women in the Oslo Area, Norway. Nutrients 2018;10(3):280; doi: 10.3390/nu10030280.
38) Hess SY, Ouédraogo CT, Young RR, et al. Urinary iodine concentration identifies pregnant women as iodine deficient yet school-aged children as iodine sufficient in rural Niger. Public Health Nutr 2017;20(7):1154-1161; doi: 10.1017/S1368980016003232.
39) Hess SY, Zimmermann MB, Torresani T, et al. Monitoring the adequacy of salt iodization in Switzerland: a national study of school children and pregnant women. Eur J Clin Nutr 2001;55(3):162-166; doi: 10.1038/sj.ejcn.1601140.
40) Hiéronimus S, Bec-Roche M, Ferrari P, et al. Iodine status and thyroid function of 330 pregnant women from Nice area assessed during the second part of pregnancy. Ann Endocrinol (Paris) 2009;70(4):218-224; doi: 10.1016/j.ando. 2009.03.004.
41) Hu LY, Zhu CH, Su MF, et al. [Urinary iodine concentration status and its influencing factors of pregnant women in Yuhuan county, Zhejiang Province]. Zhonghua Yu Fang Yi Xue Za Zhi 2018;52(1):85-87; doi: 10.3760/cma.j.issn. 0253-9624.2018.01.016.
42) Jaiswal N, Melse-Boonstra A, Sharma SK, et al. The iodized salt programme in Bangalore, India provides adequate iodine intakes in pregnant women and more-than-adequate iodine intakes in their children. Public Health Nutr 2015;18(3):403-413; doi: 10.1017/S136898001400055X.
43) Jaiswal N, Melse-Boonstra A, Thomas T, et al. High prevalence of maternal hypothyroidism despite adequate iodine status in Indian pregnant women in the first trimester. Thyroid 2014;24(9):1419-1429; doi: 10.1089/thy.2014. 0071.
44) Jaruratanasirikul S, Chukamnerd J, Koranantakul O, et al. The relationship of maternal iodine status and neonatal thyrotropin concentration: a study in Southern Thailand. J Pediatr Endocrinol Metab 2006;19(5):727-732; doi: 10.1515/jpem.2006.19.5.727.
45) Jaruratanasirikul S, Sangsupawanich P, Koranantakul O, et al. Maternal iodine status and neonatal thyroid-stimulating hormone concentration: a community survey in Songkhla, southern Thailand. Public Health Nutr 2009;12(12):2279-2284; doi: 10.1017/S1368980009005205.
46) Joshi K, Nair S, Khade C, et al. Early gestation screening of pregnant women for iodine deficiency disorders and iron deficiency in urban centre in Vadodara, Gujarat, India. J Dev Orig Health Dis 2014;5(1):63-68; doi: 10.1017/S2040174413000470.

47) Kapil U, Pandey RM, Sareen N, et al. Iodine nutritional status in Himachal Pradesh state, India. J Endocrinol Metab. 2015;19(5):602-607; doi: 10.4103/ 2230-8210.163173.
48) Kapil U, Sareen N, Nambiar VS, et al. Status of iodine nutrition among pregnant mothers in selected districts of Uttarakhand, India. J Endocrinol Metab. 2015;19(1):106-109. doi: 10.4103/2230-8210.131764.
49) Konrade I, Kalere I, Strele I, et al. Iodine deficiency during pregnancy: a national cross-sectional survey in Latvia. Public Health Nutr 2015;18(16): 2990-2997; doi: 10.1017/S1368980015000464.
50) Korenek A, Prochazka M. Iodine in early pregnancy--is there enough? Biomed Pap Med Fac Univ Palacky Olomouc Czech Repub 2008;152(1):97-99; doi: 10.5507/bp.2008.015.
51) Korevaar TI, Chaker L, Jaddoe VW, et al. Maternal and Birth Characteristics Are Determinants of Offspring Thyroid Function. J Clin Endocrinol Metab 2016;101(1):206-213; doi: 10.1210/jc.2015-3559.
52) Korevaar TIM, Schalekamp-Timmermans S, Rijke YB, et al. Hypothyroxinemia and TPO-antibody positivity are risk factors for premature delivery: the generation R study. J Clin Endocrinol Metab 2013;98(11):4382-4390; doi: 10.1210/jc.2013-2855.
53) Kung AW, Lao TT, Chau MT, et al. Goitrogenesis during pregnancy and neonatal hypothyroxinaemia in a borderline iodine sufficient area. Clin Endocrinol (Oxf) 2000;53(6):725-731; doi: 10.1046/j.1365-2265.2000. 01156.x.
54) Kung AWC, Chau MT, Lao TT, et al. The effect of pregnancy on thyroid nodule formation. J Clin Endocrinol Metab 2002;87(3):1010-1014; doi: 10.1210/ jcem.87.3.8285.
55) Kurtoğlu S, Akcakuş M, Kocaoğlu C, et al. Iodine deficiency in pregnant women and in their neonates in the central Anatolian region (Kayseri) of Turkey. Turk J Pediatr 2004;46(1):11-15.
56) Kurtoglu S, Akcakus M, Kocaoglu C, et al. Iodine status remains critical in mother and infant in Central Anatolia (Kayseri) of Turkey. Eur J Nutr 2004;43(5):297-303; doi: 10.1007/s00394-004-0474-2.
57) Kut A, Gursoy A, Senbayram S, et al. Iodine intake is still inadequate among pregnant women eight years after mandatory iodination of salt in Turkey. J Endocrinol Invest 2010;33(7):461-464; doi: 10.1007/BF03346625.
58) Lean MI, Lean ME, Yajnik CS, et al. Iodine status during pregnancy in India and related neonatal and infant outcomes. Public Health Nutr. 2014;17(6):1353-1362; doi: 10.1017/S1368980013001201.
59) Lean MI, Lean ME, Yajnik CS, et al. Iodine status during pregnancy in India and related neonatal and infant outcomes. Public Health Nutr. 2014;17(6): 1353-1362, doi: 10.1017/S1368980013001201.
60) Lee YA, Cho SW, Sung HK, et al. Effects of Maternal Iodine Status during Pregnancy and Lactation on Maternal Thyroid Function and Offspring Growth and Development: A Prospective Study Protocol for the Ideal Breast Milk

Cohort. Endocrinol Metab (Seoul) 2018;33(3):395-402; doi: 10.3803/EnM. 2018.33.3.395.

61) León G, Murcia M, Rebagliato M, et al. Maternal thyroid dysfunction during gestation, preterm delivery, and birthweight. The Infancia y Medio Ambiente Cohort, Spain. Paediatr Perinat Epidemiol 2015;29(2):113-122; doi: 10.1111/ppe.12172.
62) Li H, Wang Y, Zheng J, et al. Analysis on iodine nutritional status and thyroid function in pregnant women. Wei Sheng Yan Jiu 2012;41(4):532-535.
63) Lim KK, Chan YY, Teh CH, et al. Iodine status among pregnant women in rural Sabah, Malaysia. Asia Pac J Clin Nutr 2017;26(5):861-866; doi: 10.6133/apjcn. 092016.06.
64) Lola L. Study links iodine deficiency in pregnancy with poor cognitive outcomes in children. BMJ 2013;346:f3365; doi: 10.1136/bmj.f3365.
65) Luton D, Alberti C, Vuillard E, et al. Iodine deficiency in northern Paris area: impact on fetal thyroid mensuration. PLoS One 2011;6(2):e14707; doi: 10.1371/journal.pone.0014707.
66) Marco A, Vicente A, Castro E, et al. Patterns of iodine intake and urinary iodine concentrations during pregnancy and blood thyroid-stimulating hormone concentrations in the newborn progeny. Thyroid 2010;20(11):1295-1299; doi: 10.1089/thy.2010.0046.
67) Mateo MCG, Fernández MF, Revenga VV, et al. Assessment of iodine nutritional status and thyroxine levels in pregnant women from different geographic areas of the Castile and Leon. Endocrinol Nutr 2011;58(8):416-421; doi: 10.1016/j.endonu.2011.05.014.
68) McElduff A, McElduff P, Gunton JE, et al. Neonatal thyroid-stimulating hormone concentrations in northern Sydney: further indications of mild iodine deficiency? Med J Aust 2002;176(7):317-320; doi: 10.5694/j.1326-5377.2002.tb04431.x.
69) Meng F, Zhao R, Liu P, et al. Assessment of iodine status in children, adults, pregnant women and lactating women in iodine-replete areas of China. PLoS One 2013;8(11):e81294; doi: 10.1371/journal.pone.0081294.
70) Menon KC, Skeaff SA, Thomson CD, et al. The effect of maternal iodine status on infant outcomes in an iodine-deficient Indian population. Thyroid 2011;21(12):1373-1380; doi: 10.1089/thy.2011.0130.
71) Mezosi E, Molnar I, Jakab A, et al. Prevalence of iodine deficiency and goitre during pregnancy in east Hungary. Eur J Endocrinol 2000;143(4):479-483; doi: 10.1530/eje.0.1430479.
72) Moleti M, Trimarchi F, Tortorella G, et al. Effects of Maternal Iodine Nutrition and Thyroid Status on Cognitive Development in Offspring: A Pilot Study. Thyroid 2016;26(2):296-305; doi: 10.1089/thy.2015.0336.
73) Ojule AC, Osotimehin BO. Maternal and neonatal thyroid status in Saki, Nigeria. Afr J Med Med Sci 1998;27(1-2):57-61.

74) Olivares JL, Ortiz VA, Mayer M, et al. An approach to a sanitary and social problem: urinary iodine excretion in pregnant women from a iodine deficient region. Arch Latinoam Nutr 2009;59(4):378-382.
75) Ollero MD, Toni M, Pineda JJ, et al. Thyroid Function Reference Values in Healthy Iodine-Sufficient Pregnant Women and Influence of Thyroid Nodules on Thyrotropin and Free Thyroxine Values.Thyroid 2019;29(3):421-429; doi: 10.1089/thy.2018.0324.
76) Orito Y, Oku H, Kubota S, et al. Thyroid function in early pregnancy in Japanese healthy women: relation to urinary iodine excretion, emesis, and fetal and child development. J Clin Endocrinol Metab 2009;94(5):1683-1688; doi: 10.1210/jc.2008-2111.
77) Ozberk DI, Kutlu R, Kilinc I, et al. Effects of mandatory salt iodization on breast milk, urinary iodine concentrations, and thyroid hormones: is iodine deficiency still a continuing problem?J Endocrinol Invest 2019;42(4):411-418; doi: 10.1007/s40618-018-0930-0.
78) Pedersen KM, Börlum KG, Knudsen PR, et al. Urinary iodine excretion is low and serum thyroglobulin high in pregnant women in parts of Denmark. Acta Obstet Gynecol Scand 1988;67(5):413-416; doi: 10.3109/ 00016348809004251.
79) Peris BR, Calvo FR, Tenias JMB, et al. Iodine deficiency and pregnancy. Current situation. Endocrinol Nutr 2009;56(1):9-12. doi: 10.1016/S1575-0922(09) 70188-3.
80) Pettigrew-Porter A, Skeaff S, Gray A, et al. Are pregnant women in New Zealand iodine deficient? A cross-sectional survey. Aust N Z J Obstet Gynaecol 2011;51(5):464-467; doi: 10.1111/j.1479-828X.2011.01331.x.
81) Ren IT, Jia QZ, Zhang XD, et al. [Prevalence of thyroid function in pregnant and lactating women in areas with different iodine levels of Shanxi province]. Zhonghua Liu Xing Bing Xue Za Zhi. 2018;39(5):609-613; doi: 10.3760/cma.j. issn.0254-6450.2018.05.013.
82) Renaguli A, Luo YZ, Wang XL, et al. [Relationship between thyrotropin and urine iodine in Han and Uygur nationalities pregnancy women in People's Hospital of Xinjiang Uygur Autonomous Region]. Zhonghua Fu Chan Ke Za Zhi. 2018;53(9):595-601; doi: 10.3760/cma.j.issn.0529-567x.2018.09.003.
83) Restini LAO, Dessordi R, Ferreira SMS, et al. Assessment of thyroid function, ioduria and oxidative stress in women in the first trimester of pregnancy. Nutr Hosp 2018;35(6):1387-1393; doi: 10.20960/nh.1653.
84) Rezvanian H, Aminorroaya A, Majlesi M, et al. Thyroid size and iodine intake in iodine-repleted pregnant women in Isfahan, Iran. Endocr Pract 2002;8(1):23-28; doi: 10.4158/EP.8.1.23.
85) Riaño Galán I, Martínez PS, Díaz MPM, et al. Psycho-intellectual development of 3 year-old children with early gestational iodine deficiency. J Pediatr Endocrinol Metab 2005;18 Suppl 1:1265-1272; doi: 10.1515/jpem.2005. 18.s1.1265.

86) Rodríguez MS, San Julián CA, de Aguilar VA. Iodine intake during pregnancy: effects on thyroid function in mother and child. Endocrinol Nutr 2013;60(7):352-357; doi: 10.1016/j.endonu.2013.01.010.
87) Sánchez-Vega J, del Rey FE, Fariñas-Seijas H, et al. Inadequate iodine nutrition of pregnant women from Extremadura (Spain). Eur J Endocrinol 2008;159(4): 439-445; doi: 10.1530/EJE-08-0309.
88) Sekitani Y, Hayashida N, Takahashi J, et al. Urinary iodine concentrations of pregnant women in Ukraine. Clin Chem Lab Med 2013;51(4):811-816; doi: 10.1515/cclm-2012-0397.
89) Shi L, Tian Y, Piao J, et al. Iodine nutritional status of pregnant, lactating women and children in Linxia Region of Gansu Province. Wei Sheng Yan Jiu 2002;31(4):282-284.
90) Smyth PP, Hetherton AM, Smith DF, et al. Maternal iodine status and thyroid volume during pregnancy: correlation with neonatal iodine intake. J Clin Endocrinol Metab 1997;82(9):2840-2843; doi: 10.1210/jcem.82.9.4203.
91) Snart CJP, Keeble C, Taylor E, et al. Maternal Iodine Status and Associations with Birth Outcomes in Three Major Cities in the United Kingdom. Nutrients 2019;11(2):441; doi: 10.3390/nu11020441.
92) Soares R, Vanacor R, Manica D, et al. Thyroid volume is associated with family history of thyroid disease in pregnant women with adequate iodine intake: a cross-sectional study in southern Brazil. J Endocrinol Invest 2008;31(7):614-617; doi: 10.1007/BF03345612.
93) Stinca S, Andersson M, Herter-Aeberli I, et al. Moderate-to-Severe Iodine Deficiency in the "First 1000 Days" Causes More Thyroid Hypofunction in Infants Than in Pregnant or Lactating Women. J Nutr 2017;147(4):589-595; doi: 10.3945/jn.116.244665.
94) Threapleton DE, Snart CJP, Keeble C, et al. Maternal iodine status in a multi-ethnic UK birth cohort: Associations with child cognitive and educational development. Paediatr Perinat Epidemiol. 2021;35(2):236-246; doi: 10.1111/ppe.1271.
95) Torre EM, Alvarez ED, Artal AR, et al. Iodine nutrition in pregnant women from Oviedo area. Is iodine supplementation necessary? Endocrinol Nutr 2014;61(8):404-409; doi: 10.1016/j.endonu.2014.02.005.
96) Travers CA, Guttikonda K, Norton CA, et al. Iodine status in pregnant women and their newborns: are our babies at risk of iodine deficiency? Med J Aust2006;184(12):617-620; doi: 10.5694/j.1326-5377.2006.tb00417.x.
97) van Mil NH, Tiemeier H, Bongers-Schokking JJ, et al. Low urinary iodine excretion during early pregnancy is associated with alterations in executive functioning in children. J Nutr 2012;142(12):2167-2174; doi: 10.3945/jn.112.161950.
98) Velasco I, Martín J, Gallego M, et al. Maternal-fetal thyroid function at the time of birth and its relation with iodine intake. Thyroid 2013;23(12):1619-1626; doi: 10.1089/thy.2013.0035.

99) Vermiglio F, Lo Presti VP, Castagna MG, et al. Increased risk of maternal thyroid failure with pregnancy progression in an iodine deficient area with major iodine deficiency disorders. Thyroid 1999;9(1):19-24; doi: 10.1089/thy.1999.9.19.
100) Wang K, Zhang J, Li F, et al. Urinary iodine in early pregnancy is associated with subclinical hypothyroidism in Tianjin, China: an observational study. BMC Endocr Disord 2017;17(1):10; doi: 10.1186/s12902-017-0162
101) Wang Y, Dou Y, Zhu X, et al. A comparative study on iodine nutrition and thyroid function of different population in urban and rural areas of Wuwei, Gansu province. Zhonghua Liu Xing Bing Xue Za Zhi 2015;36(10):1095-1098.
102) Wang Y, Ge P, Ma Q, et al. Study on the relationship between iodine status and growth in infants at the key period of brain development. Zhonghua Yi Xue Za Zhi 2012;33(2):164-167.
103) Wang Y, Ge P, Wang G, et al. Study on the status of nutrition in pregnant women, lactating women and babies in Yongjing, Gansu province. Zhonghua Liu Xing Bing Xue Za Zhi 2008;29(3):258-261.
104) Wang Y, SunW, Zhu X, et al. Changes of iodine nutrition status and thyroid function among pregnant women in iodine sufficient rural area of Gansu province. Zhonghua Liu Xing Bing Xue Za Zhi 2014;35(1):49-52.
105) Wang Y, Zhang Z, Ge P, et al. Iodine status and thyroid function of pregnant, lactating women and infants (0-1 yr) residing in areas with an effective Universal Salt Iodization program Asia Pac J Clin Nutr 2009;18(1):34-40.
106) Wei Z, Wang W, Zhang J, et al. Urinary iodine level and its determinants in pregnant women of Shanghai, China. Br J Nutr 2015;113(9):1427-1432; doi: 10.1017/S0007114515000665.
107) Zhang D, Cai K, Wang G, et al. Trimester-specific reference ranges for thyroid hormones in pregnant women. Medicine (Baltimore) 2019;98(4):e14245; doi: 10.1097/MD.0000000000014245.
108) Zhang Y, Liu F, Sun W, et al. Establishment of reference ranges for thyroid-related indicators in normal pregnant women. Zhonghua Yi Xue Za Zhi 2016;96(5):339-343; doi: 10.3760/cma.j.issn.0376-2491.2016.05.005.
109) Zhou R, Tao Y, Dong X, et al. Study on the relation between iodine nutrition of pregnant women in different occasions and thyroid function of their neonates. Zhonghua Liu Xing Bing Xue Za Zhi 2002;23(5):356-359.
110) Zimmermann MB, Aeberli I, Torresani T, et al. Increasing the iodine concentration in the Swiss iodized salt program markedly improved iodine status in pregnant women and children: a 5-y prospective national study. Am J Clin Nutr 2005;82(2):388-392; doi: 10.1093/ajcn.82.2.388.
111) Zou S, Wu F, Guo C, et al. Iodine nutrition and the prevalence of thyroid disease after salt iodization: a cross-sectional survey in Shanghai, a coastal area in China. PLoS One 2012;7(7):e40718; doi: 10.1371/journal.pone. 0040718.

The reasons of the excluded studies

Study	Year	Reason
Ardawi	2002	Participants were not divided according to UIC levels (<150 µg/L versus ≥ 150 µg/L or versus 150–249 µg/L).
Azizi	2003	Participants were not divided according to UIC levels (<150 µg/L versus ≥ 150 µg/L or versus 150–249 µg/L).
Blumenthal	2012	Participants were not divided according to UIC levels (<150 µg/L versus ≥ 150 µg/L or versus 150–249 µg/L).
Brucker-Davis	2012	The outcomes evaluation was not according to UIC levels <150 µg/L versus ≥ 150 µg/L or versus 150–249 µg/L.
Castillo	2018	Participants were not divided according to UIC levels (<150 µg/L versus ≥ 150 µg/L or versus 150–249 µg/L).
Celik	2016	The authors did not present the results between UIC levels and outcomes of interest
Chakraborty	2006	Non pregnant women as control group
Chakraborty	2010	Non pregnant women as control group
Chaudhary	2017	Participants were not divided according to UIC levels (<150 µg/L versus ≥ 150 µg/L or versus 150–249 µg/L).
Chen	2015	Participants were not divided according to UIC levels (<150 µg/L versus ≥ 150 µg/L or versus 150–249 µg/L).
Chinyanga	2006	Participants were not divided according to UIC levels (<150 µg/L versus ≥ 150 µg/L or versus 150–249 µg/L).
Chinyanga	1989	Non pregnant women as control group
Costeira	2009	Participants were not divided according to UIC levels (<150 µg/L versus ≥ 150 µg/L or versus 150–249 µg/L)
Cromie	2020	Participants were not divided according to UIC levels <150 µg/L versus ≥ 150 µg/L or versus 150–249 µg/L.
Cuellar-Rufino	2017	A case-control study in pregnant women
Das	2005	Non pregnant women as control group
De Zoysa	2016	Participants were not divided according to UIC levels (<150 µg/L versus ≥ 150 µg/L or versus 150–249

		μg/L).
Dodd	1993	The outcomes evaluation was not according to UIC levels <150 μg/L versus ≥ 150 μg/L or versus 150–249 μg/L.
Dold S	2018	Participants were not divided according to UIC levels (<150 μg/L versus ≥ 150 μg/L or versus 150–249 μg/L)
Domínguez	2004	Non pregnant women as control group
Elahi	2009	Different values of UIC levels for iodine sufficient and insufficiency
Elnagar	1998	Participants were not divided according to UIC levels (<150 μg/L versus ≥ 150 μg/L or versus 150–249 μg/L).
Eltom	2000	Non outcome of Interest
Eltom	1999	Non pregnant women as control group
Farebrother	2018	Participants were not divided according to UIC levels (<150 μg/L versus ≥ 150 μg/L or versus 150–249 μg/L).
Fereja	2018	No evaluation of UIC levels and outcomes of interest
Filteau	1994	The outcomes evaluation was not according to UIC levels <150 μg/L versus ≥ 150 μg/L or versus 150–249 μg/L.
Fister	2009	Participants were not divided according to UIC levels (<150 μg/L versus ≥ 150 μg/L or versus 150–249 μg/L).
Fuse	2013	Participants were not divided according to UIC levels (<150 μg/L versus ≥ 150 μg/L or versus 150–249 μg/L).
Fuse	2011	The outcomes evaluation was not according to UIC levels <150 μg/L versus ≥ 150 μg/L or versus 150–249 μg/L.
Gaitan	1989	Case control study
Gietka-Czernel	2010	Participants were not divided according to UIC levels (<150 μg/L versus ≥ 150 μg/L or versus 150–249 μg/L).
Gowachirapant	2014	No control group; pregnant women with UIC levels ≥ 150 μg/L or versus 150–249 μg/L)
Grau	2015	Participants with hypothyroxinemia

Guan	2005	The authors did not present the results between UIC levels and outcomes of interest
Gunnarsdottir	2013	Participants were not divided according to UIC levels (<150 µg/L versus ≥ 150 µg/L or versus 150–249 µg/L).
Habimana	2013	Participants were not divided according to UIC levels (<150 µg/L versus ≥ 150 µg/L or versus 150–249 µg/L).
Henjum	2018	Participants were not divided according to UIC levels (<150 µg/L versus ≥ 150 µg/L or versus 150–249 µg/L).
Hess	2001	Participants were not divided according to UIC levels (<150 µg/L versus ≥ 150 µg/L or versus 150–249 µg/L).
Hiéronimus	2009	No evaluation of the association between UIC levels and outcomes of interest
Hu	2018	We had no access
Jaiswal	2015	Participants were not divided according to UIC levels (<150 µg/L versus ≥ 150 µg/L or versus 150–249 µg/L).
Jaiswal	2014	Participants were not divided according to UIC levels (<150 µg/L versus ≥ 150 µg/L or versus 150–249 µg/L).
Jaruratanasirikul	2006	No evaluation of the association between UIC levels and outcomes of interest
Jaruratanasirikul	2008	No evaluation of the association between UIC levels and outcomes of interest
Joshi	2014	No evaluation of the association between UIC levels and outcomes of interest
Kapil	2015	Participants were not divided according to UIC levels (<150 µg/L versus ≥ 150 µg/L or versus 150–249 µg/L).
Kapil	2015	Participants were not divided according to UIC levels (<150 µg/L versus ≥ 150 µg/L or versus 150–249 µg/L).
Konrade	2015	No evaluation of the association between UIC levels and outcomes
Korevaar	2016	No evaluation of the association between UIC levels and outcomes

Kung	2002	No evaluation of outcome of interest
Kung	2000	Participants were not divided according to UIC levels (<150 μg/L versus ≥ 150 μg/L or versus 150–249 μg/L).
Kurtoglu	2004	Participants were not divided according to UIC levels <150 μg/L versus ≥ 150 μg/L or versus 150–249 μg/L.
Kurtoğlu	2004	Participants were not divided according to UIC levels <150 μg/L versus ≥ 150 μg/L or versus 150–249 μg/L.
Kut	2010	Participants were not divided according to UIC levels <150 μg/L versus ≥ 150 μg/L or versus 150–249 μg/L.
Lean	2014	The outcomes evaluation was not according to UIC levels <150 μg/L versus ≥ 150 μg/L or versus 150–249 μg/L.
Lean	2014	The outcomes evaluation was not according to UIC levels <150 μg/L versus ≥ 150 μg/L or versus 150–249 μg/L.
Lee	2018	Study protocol
Léon	2015	Editorial
Lim	2017	The outcomes evaluation was not according to UIC levels <150 μg/L versus ≥ 150 μg/L or versus 150–249 μg/L.
Lim	2012	The outcomes evaluation was not according to UIC levels <150 μg/L versus ≥ 150 μg/L or versus 150–249 μg/L.
Lola	2013	Editorial
Luton	2011	No evaluation of outcome of interest
Marco	2010	Participants were not divided according to UIC levels <150 μg/L versus ≥ 150 μg/L or versus 150–249 μg/L.
Mateo	2011	No control group; pregnant women with UIC levels ≥ 150 μg/L or versus 150–249 μg/L)
McElduff	2002	The outcomes evaluation was not according to UIC levels <150 μg/L versus ≥ 150 μg/L or versus 150–249 μg/L.
Meng	2013	The outcomes evaluation was not according to UIC levels <150 μg/L versus ≥ 150 μg/L or versus 150–

		249 µg/L.
Menon	2011	The outcomes evaluation was not according to UIC levels <150 µg/L versus ≥ 150 µg/L or versus 150–249 µg/L.
Mezosi	2000	The outcomes evaluation was not according to UIC levels <150 µg/L versus ≥ 150 µg/L or versus 150–249 µg/L.
Moleti	2015	The outcomes evaluation was not according to UIC levels <150 µg/L versus ≥ 150 µg/L or versus 150–249 µg/L.
Ojule	1998	The outcomes evaluation was not according to UIC levels <150 µg/L versus ≥ 150 µg/L or versus 150–249 µg/L.
Olivares	2009	Participants were not divided according to UIC levels <150 µg/L versus ≥ 150 µg/L or versus 150–249 µg/L.
Ollero	2019	Participants were not divided according to UIC levels (<150 µg/L versus ≥ 150 µg/L or versus 150–249 µg/L)
Orito	2009	The outcomes evaluation was not according to UIC levels <150 µg/L versus ≥ 150 µg/L or versus 150–249 µg/L.
Ozberk	2019	No evaluation of UIC levels and outcomes of interest
Pedersen	1988	No evaluation of outcome of interest
Peris	2009	The outcomes evaluation was not according to UIC levels <150 µg/L versus ≥ 150 µg/L or versus 150–249 µg/L.
Pettigrew-Porter	2011	Participants were not divided according to UIC levels <150 µg/L versus ≥ 150 µg/L or versus 150–249 µg/L.
Ren	2018	We had no access
Renaguli	2018	We had no access
Restini	2018	Participants were not divided according to UIC levels (<150 µg/L versus ≥ 150 µg/L or versus 150–249 µg/L)
Rezvanian	2002	Participants were not divided according to UIC levels <150 µg/L versus ≥ 150 µg/L or versus 150–249 µg/L.
Riaño Galán	2005	Participants were not divided according to UIC levels <150 µg/L versus ≥ 150 µg/L or versus 150–249 µg/L.

Rodríguez	2013	The outcomes evaluation was not according to UIC levels <150 μg/L versus ≥ 150 μg/L or versus 150–249 μg/L.
Rydbeck	2009	Participants were not divided according to UIC levels (<150 μg/L versus ≥ 150 μg/L or versus 150–249 μg/L).
Sánchez-Vega	2008	Participants were not divided according to UIC levels <150 μg/L versus ≥ 150 μg/L or versus 150–249 μg/L.
Sekitani	2013	Non pregnant women as control group
Shi	2002	Participants were not divided according to UIC levels <150 μg/L versus ≥ 150 μg/L or versus 150–249 μg/L.
Smyth	1997	Non pregnant women as control group
Snart	2018	Participants were not divided according to UIC levels (<150 μg/L versus ≥ 150 μg/L or versus 150–249 μg/L).
Soares	2008	The outcomes evaluation was not according to UIC levels <150 μg/L versus ≥ 150 μg/L or versus 150–249 μg/L.
Soldin	2005	Participants were not divided according to UIC levels (<150 μg/L versus ≥ 150 μg/L or versus 150–249 μg/L).
Stinca	2017	No control group; pregnant women with UIC levels ≥ 150 μg/L or versus 150–249 μg/L)
Threapleton	2021	Participants were not divided according to UIC levels <150 μg/L versus ≥ 150 μg/L or versus 150–249 μg/L.
Torre	2014	The outcomes evaluation was not according to UIC levels <150 μg/L versus ≥ 150 μg/L or versus 150–249 μg/L.
Travers	2006	The outcomes evaluation was not according to UIC levels <150 μg/L versus ≥ 150 μg/L or versus 150–249 μg/L.
van Mil	2012	Participants were not divided according to UIC levels <150 μg/L versus ≥ 150 μg/L or versus 150–249 μg/L.
Velasco	2013	The outcomes evaluation was not according to UIC levels <150 μg/L versus ≥ 150 μg/L or versus 150–249 μg/L.
Vermiglio	2009	The control and exposure groups were from regions with different iodine supply

Wang	2017	Case control study
Wang	2015	The outcomes evaluation was not according to UIC levels <150 μg/L versus ≥ 150 μg/L or versus 150–249 μg/L.
Wang	2009	The outcomes evaluation was not according to UIC levels <150 μg/L versus ≥ 150 μg/L or versus 150–249 μg/L.
Wang	2012	The outcomes evaluation was not according to UIC levels <150 μg/L versus ≥ 150 μg/L or versus 150–249 μg/L.
Wang	2014	Participants were not divided according to UIC levels <150 μg/L versus ≥ 150 μg/L or versus 150–249 μg/L.
Wang	2008	Participants were not divided according to UIC levels <150 μg/L versus ≥ 150 μg/L or versus 150–249 μg/L.
Wei	2015	The outcomes evaluation was not according to UIC levels <150 μg/L versus ≥ 150 μg/L or versus 150–249 μg/L.
Zhang	2019	Non-pregnant women as control group
Zhang	2016	The outcomes evaluation was not according to UIC levels <150 μg/L versus ≥ 150 μg/L or versus 150–249 μg/L.
Zhou	2002	Participants were not divided according to UIC levels <150 μg/L versus ≥ 150 μg/L or versus 150–249 μg/L.
Zimmermann	2005	No evaluation of outcome of interest
Zou	2012	The outcomes evaluation was not according to UIC levels <150 μg/L versus ≥ 150 μg/L or versus 150–249 μg/L.
